# Dual‐Targeting, Hybrid Antifungal Agents Targeting *Candida albicans*—An Overview

**DOI:** 10.1111/cbdd.70380

**Published:** 2026-08-21

**Authors:** Sarmistha Pal, Sriparna Pusti, Iftakhar Hussain Ansari, Sanchita Pradhan, Hritabrata Bhattacharya, Shuvradip Das

**Affiliations:** ^1^ Department of Pharmaceutical Technology JIS University Kolkata India

**Keywords:** *Candida albicans*, candidiasis, CYP51 inhibition, fungicidal agents, hybrid antifungals

## Abstract

Due to environmental degradation, substance abuse, and immunological loss, fungal diseases are rising. *Candida* sp. can inflame tissues and organs. In addition, fungi enter deep tissues to generate invasive fungal infections (IFIs), a long‐term worldwide health risk, especially for HIV/chemotherapy patients with compromised immune systems. The present treatment uses single‐target antifungals like azoles, allylamines, and pyrimidines. But drug‐resistant fungi and widespread adverse effects have limited their therapeutic efficacy. Thus, innovative, safe, and effective antifungals are needed. Through the 20th century, the pharmaceutical industry adopted the “one molecule, one target, one disease” mentality which led to various effective selective drugs that will dominate disease treatment. But, unfortunately, even with the best research, targeting one target may not work in complicated illnesses. Thus, drugs that activate several targets simultaneously, to improve efficacy and safety, are gaining interest. Multitarget medicines involve monotherapies, medication cocktails, and combination drugs. The second method, multitarget‐directed ligands (MTDLs), uses one active component that treats multifactorial diseases. In MTDL, new chemical entities (NCEs) are created which may solve various issues related to combination therapies, including: (1) establishing balanced and multi‐selective potency across targets, (2) matching PK/PD properties for components, (3) better patient compliance, (4) straightforward approval from regulatory authorities, (5) higher cost of medication etc. To optimize developability, effectiveness, and safety, multitarget drug design should be more methodical and thorough in target combinations, ligand selection, and desired activity equilibrium. In this review, the hybrid, dual‐targeting antifungal agents are classified into four major categories, based on the combination of their mechanisms, namely—(**1**) Antifungal + antifungal (involves dual mode of DNA damage + membrane polarization), (**2**) Antifungal + Resistance reversal (includes proteasome inhibition, Coumarin + CYP51 inhibitors, CYP51 + Hsp90 inhibitors), (**3**) Antifungal + Host immunity modulators (includes CYP51 + HDAC inhibitors, NLRP3 inflammasome + AHAS inhibitors, COX + CYP51 inhibitors, PD‐L1 + CYP51 inhibition etc.), (**4**) Azole sensitisers (JAK2 + HDAC inhibition, BRD4 + HDAC inhibition etc.), (**5**) Multifunctional, miscellaneous antifungal agents (DNA intercalation + intracellular ROS production) etc. Thus, it covers significant achievements and the associated challenges of antifungal multitarget drug design, which might be helpful to the researchers who are interested in delving into the development of fungicidal agents as a drug to address the burning question of drug‐resistant *Candida albicans* and invasive fungal infections (IFIs).

## Graphical abstract

The selective, single‐targeting antifungal agents serve as the parent molecule(s) for the design of dual‐targeting, hybrid fungicidal agents and are discussed and compared, which enables the researchers to understand the logic for designing multi‐targeting new chemical entities (NCEs) that can exhibit dual‐sword attack on *Candida albicans* .

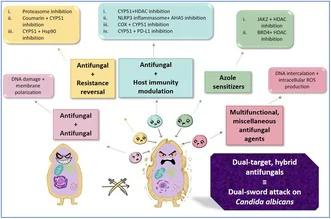

**Abbreviations**
AHAS: acetohydroxyacid synthase
Bch: terephthalic dihydrazide
BET: bromodomain and extra‐terminal
BRD4: bromodomain containing protein 4
Bt: 1,3,5‐Benzenetrialdehyde
*C. alb*.:
*Candida albicans*

*C. albicans*

:
*Candida albicans*

*C. auris*

: *Candida auris*
*C. gla*.: *Candida glabrata*

*C. glabrata*

: *Candida glabrata*
*C. kru*.:
*Candida krusei*

*C. neo*.:
*Cryptococcus neoformans*

CLSI: Clinical & Laboratory Standards Institute
COF: covalent organic framework
COX‐2: cyclooxygenase
CYP51: lanosterol 14*α*‐demethylase
FICI: fractional inhibitory concentration index
FLC: fluconazole
GSH: glutathione
GSSG: oxidized glutathione (GSSG) ratio
HNs: hydrazylnaphthalimidols
Hsp90: heat shock protein 90
IC: invasive candidiasis
IFIs: invasive fungal infections
JAK: Janus kinase
MFCs: minimum fungicidal concentrations
MIC: minimal inhibitory concentrations
MIC: minimum inhibitory concentration
MTDLs: multitarget‐directed ligands
NA: naphthalimide
OD: optical density
PD‐L1: programmed death ligand 1
PEG: Polyethylene glycols
ROS: reactive oxygen species
SI: selectivity index
Tp: 2,4,6‐trihydroxybenzene‐1,3,5‐tricarbaldehyde
ZBG: zinc‐binding group

## 1 Introduction

**FIGURE 1:**
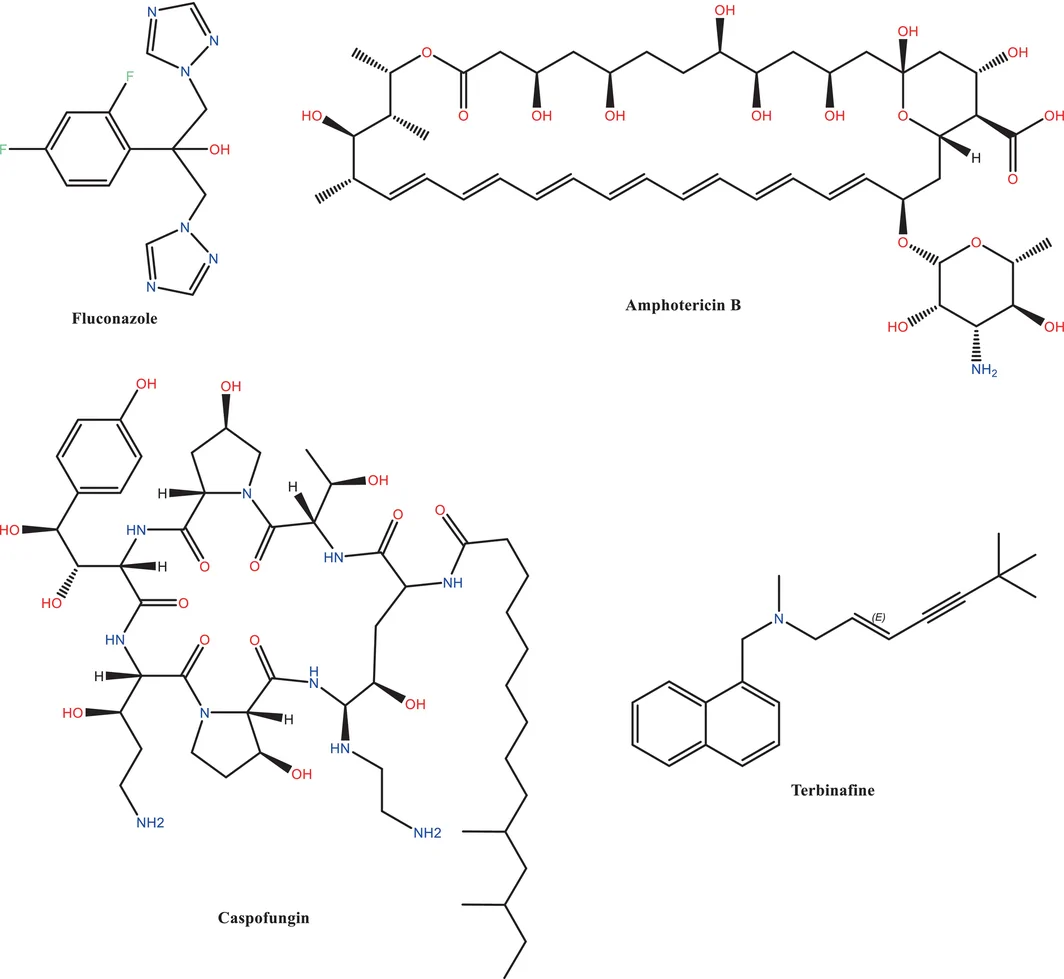
The existing single‐target antifungal agents such as azoles, allylamines, macrocyclic polyenes, lipopeptide.

The rate of fungal infections is on the rise, driven by environmental degradation, substance misuse, and declining immune function (Werkneh et al. 2022). Every year, over 1,565,000 people contract invasive candidiasis or a bloodstream infection caused by *Candida*, which results in 995,000 fatalities (63.6%) (Denning 2024; Case et al. 2025; Denning and Bromley 2015). In consultation with the WHO Advisory Group on FPPL (WHO AG FPPL), the fungal priority pathogens list (FPPL) was created to identify the most notorious fungal pathogens, nineteen fungal pathogens were included, ranked and divided into three priority groups: 1. Critical group, 2. High group, and 3. Medium group. Along with three other microorganisms— *Candida albicans* was placed in the Critical group (World Health Organization 2022) (Howard et al. 2020; Bongomin et al. 2017). These Critical group fungal strains have the capability to infiltrate the body's internal tissues and organs, leading to significant inflammatory responses (Tu et al. 2022; Chang et al. 2017). The existing treatment pathway is predominantly dependent on a narrow range of single‐target antifungal agents, such as azoles (e.g., Fluconazole), allylamines (e.g., Terbinafine), macrocyclic polyenes (e.g., Amphotericin B), lipopeptide (e.g., Caspofungin) etc. (Figure 1) (Gamaletsou et al. 2018; Seyedmousavi et al. 2017). Nonetheless, the ongoing rise of drug‐resistant fungal strains, along with certain prevalent adverse reactions, has diminished their effectiveness in clinical therapy (Mohr et al. 2011). Consequently, there is a pressing need to create innovative antifungal medications that possess both high efficacy and safety profiles.

Invasive fungal infections (IFIs) occur when fungi infiltrate deep tissues, leading to a prolonged illness that is increasingly recognized as a significant global health concern (Neoh et al. 2023; Enoch et al. 2017) (Jampilek 2016). IFIs typically arise in patients with compromised immune systems, such as individuals infected with HIV and those undergoing treatment with chemotherapeutic agents (Pathakumari et al. 2020; Zhang et al. 2023).

Nonetheless, the prolonged use of antifungal medications often results in the development of fungal resistance, and the rise of drug‐resistant fungi poses a significant challenge to global health (Cui et al. 2022). Consequently, it was anticipated that new antifungal agents exhibiting activity against drug‐resistant strains would be developed to address the clinical demand.

The principle of “one molecule, one target, one disease” prevailed in the pharmaceutical sector throughout the 20th century (Medina‐Franco et al. 2013). A multitude of effective selective pharmaceuticals has emerged from this principle, which is poised to dominate the treatment of specific diseases in the future (Hopkins 2008). In the context of multifactorial diseases, the manipulation of a singular target does not invariably yield satisfactory efficacy, even in light of the most diligent research endeavors (Costantino and Barlocco 2012). Consequently, there is a growing fascination with the development of therapeutics that concurrently engage multiple targets to enhance therapeutic efficacy and/or safety.

Multitarget therapeutics can be understood through two distinct methodologies: the first involves a blend of monotherapies, which encompasses drug cocktails (combinations of drugs each containing a single active ingredient) and combination drugs (formulations that incorporate multiple active ingredients). The second methodology focuses on multitarget‐directed ligands (MTDLs), characterized by a single tablet containing one active ingredient (Zimmermann et al. 2007).

MTDLs serve as agents that address multifactorial diseases through their engagement with various targets implicated in the pathogenesis (Bolognesi et al. 2007). This approach has the potential to circumvent numerous challenges, such as (1) the difficulty of attaining a balanced and multiselective potency across various targets, (2) it is essential to ensure that the pharmacokinetic and pharmacodynamic (PK/PD) properties of the various components are in alignment, (3) challenging to attain sequenced delivery at the intended targets (Lötsch and Geisslinger 2011; Anighoro et al. 2014; Smith et al. 2014). The merits of the aforementioned methodologies are: (1) they represent a novel chemical entity; (2) it is possible to attain a methodical administration or to modify the target exposure accordingly; (3) the development and regulatory approval processes are standardized, akin to those for single‐target drugs; (4) clinical trials could potentially progress at an accelerated pace owing to accumulated clinical experience, while the associated treatment costs might also be reduced; (5) the formulation of PK/PD properties is more straightforward when dealing with a singular active component rather than a composite mixture; (6) The therapeutic efficacy may be enhanced through synergies, even when administered at low dosages; (7) diminished negative consequences facilitate broader therapeutic ranges (Zimmermann et al. 2007).

**FIGURE 2:**
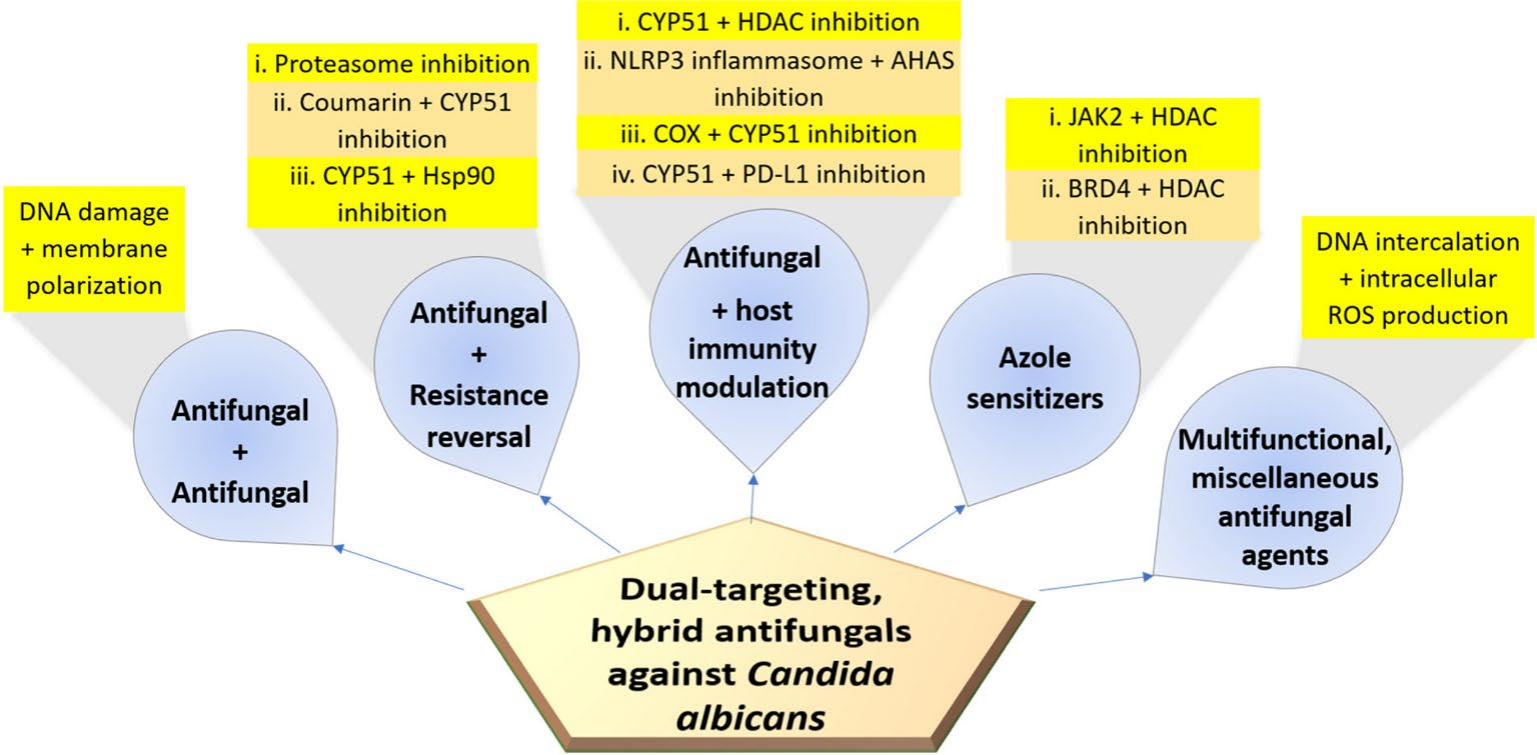
Various types of hybrids, dual‐targeting antifungal agents, based on their biological targets.

It is our belief that the design of multitarget drugs ought to be approached with a more systematic and comprehensive perspective on the interplay of target combinations, the selection of ligands, and the equilibrium of desired activities to optimize developability, efficacy, and safety. This review addresses significant issues, examines the design of antifungal multitarget drugs, and surveys key references regarding MTDLs published in recent years, aiming to offer a thorough compilation of the rational design of MTDLs (Figure 2).

Based on the combination of the target, the hybrid, dual‐ or multi‐targeting antifungal agents are broadly classified into four categories, that is, **1**. Antifungal + antifungal combination, **2**. Antifungal + Resistance reversal, **3**. Antifungal + Host immunity modulators **4**. Azole sensitizers, **5**. Multifunctional, miscellaneous antifungal agents (DNA intercalation + intracellular ROS production) etc. (Table 1).

**TABLE 1:** Mechanism‐based classification of hybrid, dual‐ or multi‐targeting antifungal agents, along with their potency, test strain, in vivo model and the primary literature (reference).

Category	Dual‐target pair	Compound code	Potency	Test strain	In vivo model	References
Antifungal + Antifungal combination	DNA damage + membrane polarization	**(Gly**_**0.8**_ **Nap**_**0.2**_ **)**_**20**_	MIC – 3.13 μg/mL; MFC—6.3 μg/mL	*C. albicans* K1	4–6‐Week‐old, female CR mice weighing 22–25 g was immunosuppressed by intraperitoneal injection of cyclophosphamide (200 mg kg^−1^), 3 days before infection and infected by intravenous injection of 200 μL *Candida albicans* suspension in saline (5 × 10^4^ CFU ml^−1^)	Zhou et al. (2024)
		**TPY**	MIC—2 μM; MFC—4 μM	*C. albicans* (SC5314)	Therapeutic efficiency of **TPY** & **TPZ** was assessed in a mouse model of *C. albicans* ‐induced vaginitis because of their strong antifungal properties and superior selectivity. Estradiol (0.3 mg in 100 μL sesame oil) was subcutaneously injected for 3 days in a row to simulate clinical settings. This resulted in vulvar edema and increased discharge, indicating a vulnerability to fungal infection. Next, 2.5 × 10^8^ CFUs of *Candida albicans* were injected intravaginally into mice. To treat the local infection, **TPY** and **TPZ** were given intravaginally 3 days after infection	Liu, Li, et al. (2024)
		**TPZ**	MIC – 2 μM; MFC—2 μM	*C. albicans* (SC5314)		
Antifungal + Resistance reversal	Proteasome inhibition	**Tat‐A**	MIC—31.25 μM	*C. albicans* SC5314	—	(Patel et al. 2020)
		**Tat‐B**	MIC—15.62 μM	*C. albicans* SC5314	—	
	Coumarin + CYP51 inhibition	**A32**	MIC—1 μg/mL	Strain CaR, fluconazole‐resistant strain of *C. albicans*	Model of candidiasis infection in mice was established by tail vein injection of *Candida albicans SC5314* and fluconazole‐resistant *Candida albicans* strain 904	(Yan et al. 2023)
			MIC—4 μg/mL	Strain 17#, fluconazole‐resistant strain of *C. albicans*		
			MIC—4 μg/mL	Strain 632, fluconazole‐resistant strain of *C. albicans*		
			MIC—4 μg/mL	Strain 901, fluconazole‐resistant strain of *C. albicans*		
			MIC—1 μg/mL	Strain 904, fluconazole‐resistant strain of *C. albicans*		
	CYP51 + Hsp90 inhibition	**MM4**	MIC_80_–0.063 μg/mL	*C. albicans* SC5314	In order to reduce their immunological response, female ICR mice (18–22 g, 4–6 weeks) received an intraperitoneal injection of cyclophosphamide (100 mg/kg) 24 h before infection. Mice received a tail vein injection of *C. albicans* SC5314 (5 × 10^5^ CFU/mouse)	Zhang et al. (2025)
Antifungal + host immunity modulation	CYP51 + HDAC inhibition	**12 h**	MIC_80_–4 μg/mL	Azole sensitive *C. alb*. SC5314	Azole‐resistant *C. albicans* strain 0304103 infectious murine model	Han et al. (2020)
			MIC_80_–2 μg/mL	Azole resistant *C. alb*. 0304103		
		**15j**	MIC_80_–0.125 μg/mL	Azole sensitive *C. alb*. SC5314	Azole‐resistant *C. albicans* strain 0304103 infectious murine model	
			MIC_80_–0.25 μg/mL	Azole resistant *C. alb*. 0304103		
	NLRP3 inflammasome + AHAS inhibition	**10**	MIC_50_ ± SD—6.4 ± 2.6 μM	*C*. *albicans* SC531	—	Lowes et al. (2021)
	COX + CYP51 inhibition	**1**	MIC—0.003 μg/mL	*C. albicans* SC5314	—	Elias et al. (2022)
			MIC—0.003 μg/ML	*C. albicans* 90,028		
			MIC—0.007 μg/mL	*C. albicans* 24,433		
			MIC—0.003 μg/mL	*C. albicans* P‐87		
			MIC—0.003 μg/mL	*C. albicans* SN152		
			MIC—64 μg/mL	*C. albicans erg3Δ/Δ erg11Δ/Δ*		
		**5**	MIC—0.007 μg/mL	*C. albicans* SC5314	—	
			MIC—0.015 μg/mL	*C. albicans* 90,028		
			MIC—0.03 μg/mL	*C. albicans* 24,433		
			MIC—0.015 μg/mL	*C. albicans* P‐87		
			MIC—0.007 μg/mL	*C. albicans* SN152		
			MIC—64 μg/mL	*C. albicans erg3Δ/Δ erg11Δ/Δ*		
		**10a‐2**	MIC_50_–0.125 μg/mL	*Candida albicans* (ATCC SC5314)	*C. alb*. ATCC SC5314, 1 × 10^6^ CFU/mL, 0.1 mL injected into the abdomen region of mice twice each day to prepare the model for in vivo testing	Liu, Liu, Fan, et al. (2022)
		**16b‐3**	MIC_50_–0.25 μg/mL	*Candida albicans* (ATCC SC5314)		
		**5a‐1‐2**	MIC_50_–0.25 μg/mL	*Candida albicans* (ATCC SC5314)	*C. alb*. (1 × 10^6^ CFU/mL, 0.1 mL) was prepared and injected into the abdomen region of mice twice daily	Liu, Liu, Yu, et al. (2022)
		**14b‐1‐2**	MIC_50_–0.125 μg/mL	*Candida albicans* (ATCC SC5314)		
		**14c‐2‐1**	MIC_50_–0.125 μg/mL	*Candida albicans* (ATCC SC5314)		
	CYP51 + PD‐L1 inhibition	**L11**	MIC_50_–0.5 μg/mL	*Candida albicans* (ATCC SC5314)	BALB/c mice (Male, 18) were injected subcutaneously with a suspension of *C. alb*. SC5314 (1 × 10^5^ CFU/mL) was to construct the mouse models of fungal infection	Sun et al. (2023)
		**L20**	MIC_50_–0.25 μg/mL	*Candida albicans* (ATCC SC5314)		
		**L21**	MIC_50_–0.25 μg/mL	*Candida albicans* (ATCC SC5314)		
		**10c‐1**	MIC_50_–0.25 μg/mL	*Candida albicans* (ATCC SC5314)	—	Liu et al. (2023)
		**14a‐2**	MIC_50_–0.125 μg/mL	*Candida albicans* (ATCC SC5314)	—	
		**17c‐2**	MIC_50_–0.125 μg/mL	*Candida albicans* (ATCC SC5314)	—	
		**PEG‐Ba@Bt‐Bch@14a‐2** ^b^	—	—	Mouse models were constructed—BALB/c mice (Male, 18) infected with *Candida albicans* Strain 901 suspension (1 × 10^5^ CFU/mL)	
		**10c‐1**	MIC_50_–0.25 μg/mL	*Candida albicans* (ATCC SC5314)	—	Yu et al. (2024)
		**17b‐1**	MIC_50_–0.25 μg/mL	*Candida albicans* (ATCC SC5314)		
		**18b‐2**	MIC_50_–0.25 μg/mL	*Candida albicans* (ATCC SC5314)	The deep and shallow fungal infection model was established by injection of *C*. *alb*. 901 suspension (2 × 10^5^ CFU/mL)	
		**PEG‐HB@** **Tp‐Bch@18b‐2**	—	—		
		**9a‐2**	MIC_50_–0.25 μg/mL	*C*. *albicans* ATCC SC5314	—	Liu, Yu, et al. (2024)
		**12b‐1**	MIC_50_–0.125 μg/mL	*C*. *albicans* ATCC SC5314	—	
		**12a‐2**	MIC_50_–0.25 μg/mL	*C*. *albicans* ATCC SC5314	Mouse models with deep and shallow fungal infections were established by intravenous and subcutaneous injection with a suspension of *C. alb*. 901 (1 × 10^5^ CFU/mL)	
		**Nc‐12a‐2**	—	*C*. *albicans* ATCC SC5314		
Azole sensitizers (lacks any standalone activity against *Candida albicans* )	JAK2 + HDAC inhibition	**20a**	MIC_50_–4 μg/mL **20a** + 0.5 μg/mL FLC (FICI^a^—0.07)	*C. albicans* strain 0304103	Immunocompromised ICR mice were injected via tail vein with 15 × 10^4^ cells of FLC‐resistant *C*. *albicans* (strain no. 0304103)	Huang et al. (2018)
			MIC_50_–4 μg/mL **20a** + 0.5 μg/mL FLC (FICI—0.07)	*C. albicans* Strain 100		
			MIC_50_–8 μg/mL **20a** + 1 μg/mL FLC (FICI—0.14)	*C. albicans* strain 0710922		
	BRD4 + HDAC inhibition	**13c**	MIC_80_ 0.5 μg/mL + 4 μg/mL FLC (FICI—0.070)	azole‐resistant *C. albicans* 0304103	Mice candidiasis model was established by injecting *C. albicans* strain 0304103 cells (3 × 10^5^) into the tail vein	Huang et al. (2023)
		**17b**	MIC_80_ 8 μg/mL + 8 μg/mL FLC (FICI—0.25)			
		**B2**	MIC_80_–2 μg/mL **B2** + 2 μg/mL FLC (FICI—0.063)	*C. albicans* 0304103	Candidiasis mouse models were established in ICR mice by injection of *C*. *albicans* 0304103 cells (5 × 10^5^ per mouse) via the tail vein	Li, Huang, et al. (2023)
			MIC_80_–4 μg/mL B2 + 0.5 μg/mL FLC (FICI—0.07)	*C. albicans:* 9172		
			MIC_80_–2 μg/mL **B2** + 1 μg/mL FLC (FICI—0.047)	*C. albicans:* 10061		
			MIC_80_–4 μg/mL **B2** + 0.25 μg/mL FLC (FICI—0.19)	*C. albicans:* 9884		
Multifunctional, miscellaneous antifungal agents	DNA intercalation + intracellular ROS production	**4a**	MIC—0.001 mM	*Candida albicans* (clinical isolation)	—	Wang et al. (2024)
			MIC—0.002 mM	*Candida albicans* ATCC 90023	—	

^a^ Fractional inhibitory concentration index (FICI) is defined as the sum of the ratio that dividing the MIC_80_ value of each drug used in combination by the value of the drug used alone. Synergism and antagonism were defined by an FICI of ≤ 0.5 and > 4, respectively. A FICI between 0.5 and 4 is considered irrelevant.

^b^ 1,3,5‐Benzenetrialdehyde (Bt, 1.0 equiv), terephthalic dihydrazide (Bch, 1.1 equiv).

The first category of dual‐targeting antifungals exerts its lethal action on *C. albicans* by both dual mode of DNA damage and membrane polarization. In this category, only a limited number of antifungal agents are known, such as **(Gly**_**0.8**_ **Nap**_**0.2**_ **)**_**20**_ and **TPY**, **TPZ** etc. These ligands show appreciable MIC and/or MFCs against *C. albicans* and show encouraging results in murine models.

The second category of ligand are capable of producing both fungicidal effect and resistance reversal. It includes the target combinations like Coumarin + CYP51 inhibitors (**A32**), CYP51 + Hsp90 inhibitors (**MM4**), proteasome inhibition (**Tat‐A** & **Tat‐B**) etc. Among several antifungal candidates under this several category **MM4** seems to be promising, due to its the low MIC_50_ and results obtained from mice model infected with *C. albicans* SC5314 (most commonly tested strain of *C. albicans* ).

The next category of hybrid anti‐fungal is more prevalent, compared to the other four categories and exhibits an interesting combination of two types of effects, that is, fungicidal effect and simultaneous modulation of the host immunity. For example, CYP51 + HDAC inhibitors (**12h** & **15j**), NLRP3 inflammasome + AHAS inhibitors (**10**), COX + CYP51 inhibitors (hybrid **1** & **5**, **10a‐2**, **16b‐3**, **14b‐1‐2**, **14c‐2‐1**), PD‐L1 + CYP51 inhibition (**L20**, **L21**, **14a‐2**, **17c‐2**, **10c‐1**, **17b‐1**, **18b‐2**, **9a‐2**, **12b‐1**, **12a‐2**) etc. In this category, the dual inhibition of PD‐L1 and CYP51 is reported several times showing promising result, although the modulation of PD‐L1 is tricky as it serves as the checkpoint in the host immune system.

The fourth category is called azole sensitizers/adjuvants as they fail to produce any fungicidal effect even at high concentrations and only show good synergistic activity when used in combination with azole antifungals (e.g., Fluconazole). Dual‐targeting ligands like JAK2 + HDAC inhibition (**20a**), BRD4 + HDAC inhibition (**13c**, **17b**, **B2**) etc. come under the category of azole sensitizers.

There are multifunctional, miscellaneous antifungal agents (**4a**), which show DNA intercalation along with intracellular ROS production. CRISPR‐Cas9 antifungals are also a promising emerging strategy to combat the dreadful rise of drug‐resistant fungal infection.

## 2 Hybrid Antifungals

### 2.1 Antifungal + Antifungal Combination

#### 2.1.1 DNA Damage + Membrane Depolarization

DNA binding investigations utilizing the competitive displacement of (**Gly**_**0.8**_ **Nap**_**0.2**_)_**20**_ against diverse DNA–dye complexes, along with the zeta potential assay, collectively suggested various interaction modalities between (**Gly**_**0.8**_ **Nap**_**0.2**_)_**20**_ and fungal DNA, encompassing minor groove binding, intercalation binding, and electrostatic interactions. Various binding modalities resulted in fungal DNA damage, significantly affecting the powerful antifungal efficacy of (**Gly**_**0.8**_ **Nap**_**0.2**_)_**20**_. Furthermore, the disruption of the fungal membrane suggested that the naphthalene moiety significantly contributed as the hydrophobic component facilitating hydrophobic–hydrophobic interactions between (**Gly**_**0.8**_ **Nap**_**0.2**_)_**20**_ and the fungal membrane.

**FIGURE 3:**
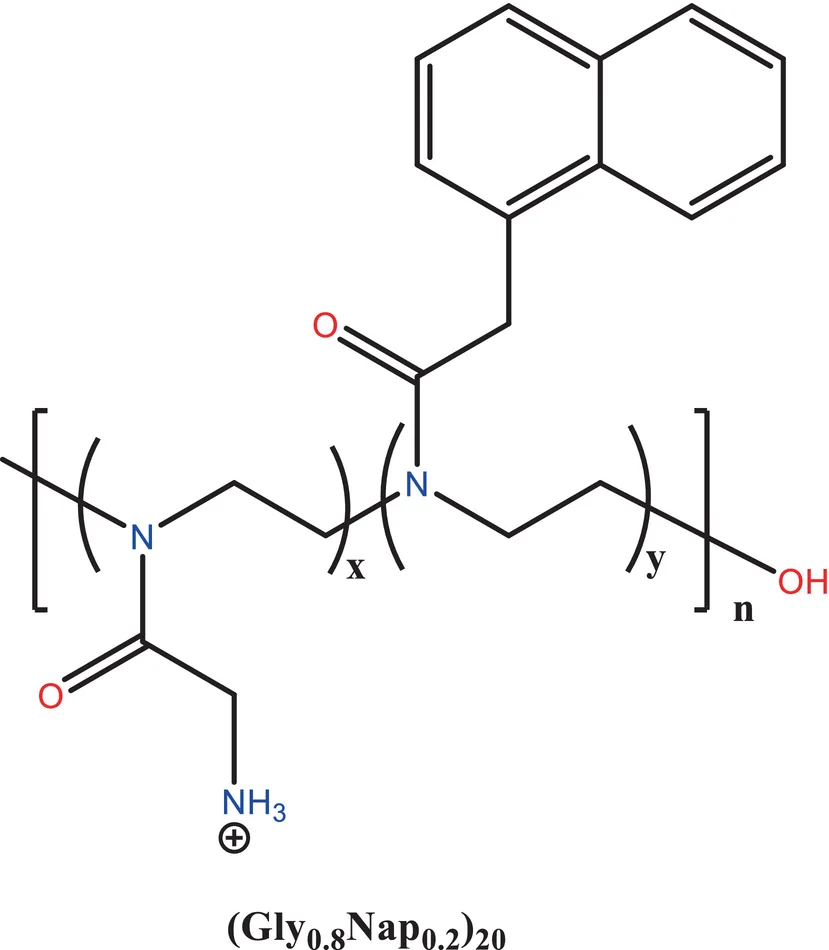
(**G** **ly**_**0.8**_ **Nap**_**0.2**_)_**20**_, that eradicate fungus by mechanisms targeting membranes and DNA.

**(Gly**_**0.8**_ **Nap**_**0.2**_ **)**_**20**_ (Figure 3) has been confirmed to successfully eradicate fungus by mechanisms targeting membranes and DNA. **(Gly**_**0.8**_ **Nap**_**0.2**_ **)**_**20**_ demonstrates significant and selective antifungal efficacy against drug‐resistant *Candida species* and other pathogenic fungal species, encompassing various clinically isolated strains. This poly(2‐oxazoline) exhibits notable therapeutic activity against drug‐resistant *Candida albicans* infections in models of cutaneous abrasion, keratitis, and systemic infection, while demonstrating no tissue or systemic damage. Furthermore, the dual‐targeting antifungal **(Gly**_**0.8**_ **Nap**_**0.2**_ **)**_**20**_ did not promote antifungal resistance (Zhou et al. 2024).

The design strategy investigated the potential of dual targeting of membranes and DNA in antifungal development, offering a promising option to combat the growing issue of antifungal resistance.

The efficacy of this technique will stimulate contemplation and discourse over the potential of targeting both fungal DNA and cell membranes to resolve the existing challenges in antifungal drug development. Moreover, it is essential to investigate thoroughly the design of advanced dual‐mechanism antifungal agents that target both membrane and DNA. The dual‐targeting strategy facilitated the development and investigation of selective antifungal drugs, providing a viable method to address drug resistance and battle drug‐resistant fungal infections.

This study possesses many possible drawbacks. The development of dual‐targeting antifungal agents as prospective therapeutic candidates necessitates a comprehensive biosafety evaluation. While initial toxicity tests have been performed in this research, additional animal studies and long‐term assessments of possible toxicity will yield further biosafety data. Secondly, the processes by which chemicals infiltrate the fungal membrane are a critical aspect of the dual‐mechanism antifungal activity and warrant comprehensive investigation in the future. The structural properties of dual‐targeting antifungal drugs may affect their interaction with fungal DNAs, requiring the investigation of a more effective design to improve DNA‐targeting antifungal efficacy. Exploring the membrane‐penetrating mechanism and improving DNA binding affinities of dual‐targeting antifungal agents are crucial avenues for next study.

**FIGURE 4:**
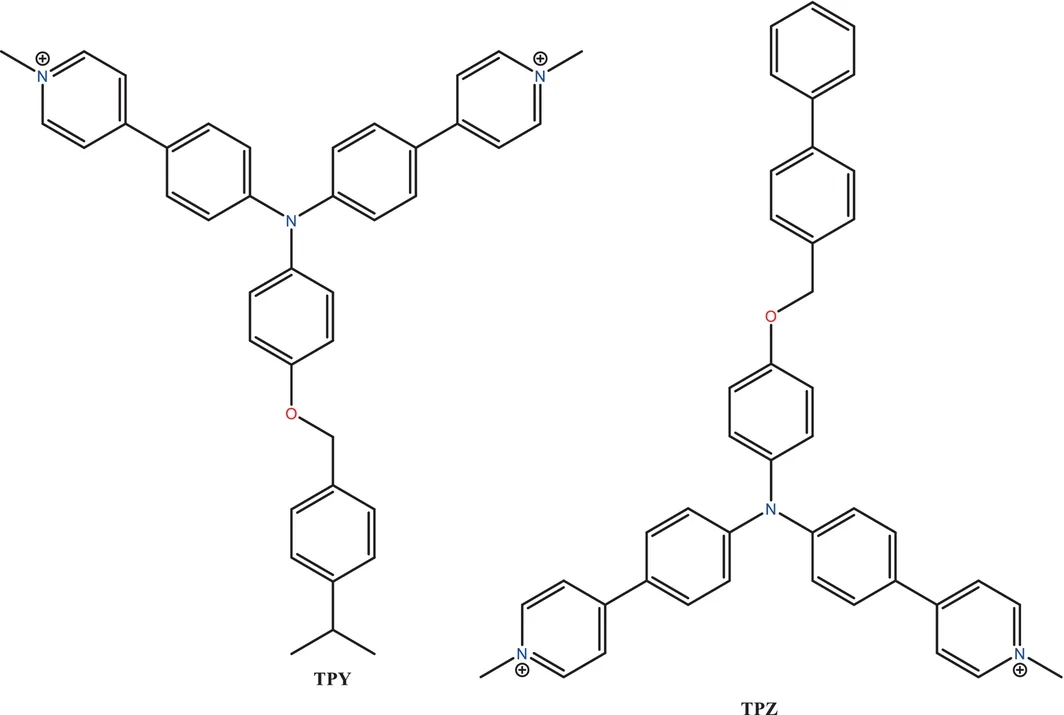
**TPY** and **TPZ**, which demonstrate appreciable membrane penetration and DNA‐binding abilities in *Candida albicans* .

Resistance to antifungal medications is a significant issue, necessitating novel therapeutic approaches. The dual‐targeting mechanism of action (MoA) has been substantiated as an effective technique for mitigating drug resistance in the development of antibacterial medicines. The structural similarities between mammalian and fungal cells hinder the development of antifungal strategies, as selectivity may be impaired. Bin Liu and his team provided a dual‐targeting method that targets fungal infections by specifically delivering DNA‐binding compounds into fungal nuclei. They integrated rigid hydrophobic units into a DNA‐binding domain to create antifungal luminogens **TPY** and **TPZ** (Figure 4), which demonstrate improved membrane penetration and DNA‐binding abilities. These chemicals demonstrate a dual‐targeting mechanism of action by depolarizing fungal membranes and producing DNA damage, hence enhancing their efficacy against fungal infections exhibiting no observable treatment resistance. **TPY** and **TPZ** revealed significant antifungal activity in vitro and displayed optimal therapeutic efficiency in a mouse model of *C. albicans* ‐induced vaginitis. This comprehensive strategy shows potential for surmounting medication resistance and enhancing antifungal treatment (Liu, Li, et al. 2024).

This study involved the design and synthesis of two dual‐targeting luminous chemicals, **TPY** and **TPZ**, both derived from the same DNA‐binding domain. This design selectively incorporates **TPY** and **TPZ** into fungal nuclei while maintaining the integrity of human cell nuclei (Figure 4). The integration of isopropylbenzene and biphenyl moieties into the DNA‐binding domain significantly improved the infiltration of both luminogens into fungal cells and nuclear membranes, while also providing a mechanism of action on fungal membranes.

Thus, **TPY** and **TPZ** demonstrate dual modes of action. Both compounds exhibited significant fungicidal action by depolarizing fungal membranes and causing DNA damage while exhibiting negligible toxicity to mammalian cells.

Additionally, **TPY** and **TPZ** demonstrated significant therapeutic activity against *C. albicans* infections in the vaginitis murine model. This dual‐targeting strategy presents an innovative method for creating effective antifungal medicines to address fungal infections and reduce antifungal resistance.

### 2.2 Antifungal + Resistance Reversal

#### 2.2.1 Proteasome Inhibitors

The ubiquitin‐26S proteasome pathway in fungal strains like *S. cerevisiae* and *C. albicans* closely resembles the mammalian process, with these enzymes playing a role in the pathogenicity of fungal species (Murray et al. 2000; Liu and Xue 2011). Consequently, targeting the ubiquitin‐proteasome pathway holds potential for the development of innovative antifungal agents and offers a viable alternative to the traditional antimicrobial mechanism of membrane lysis against human fungal pathogens like *C. albicans* , whose biofilms are challenging to treat.

**FIGURE 5:**
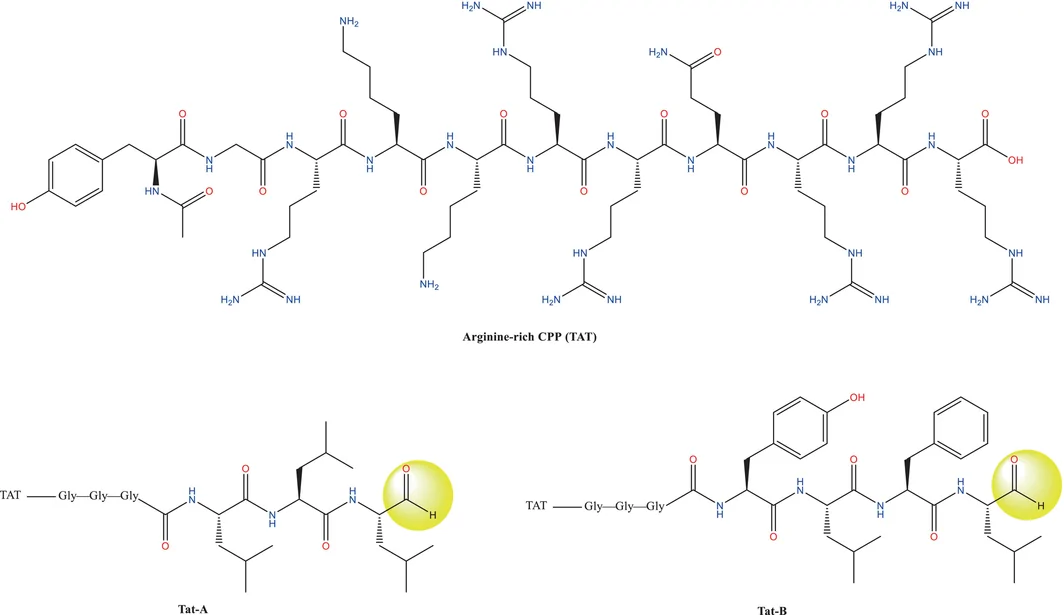
Chemical structure of peptide aldehyde **Tat‐A** and **Tat‐B**.

Peptide aldehydes linked with cell‐penetrating peptides **Tat‐A** and **Tat‐B** (Figure 5) exhibited low micromolar anticancer and antifungal efficacy, demonstrating synergistic effects when combined with cisplatin and amphotericin B against cancerous and fungal cells, respectively. **Tat‐A** and **Tat‐B** exhibited markedly greater potency than Ixazomib in inhibiting human 20S proteasomes, with IC_50_ values in the low nanomolar range. Treatment with **Tat‐A** and **Tat‐B** resulted in membrane breakdown and pore formation in HeLa and BE(2)‐C cells, as well as the suppression and eradication of *C. albicans* biofilms (Patel et al. 2020).

The TAT conjugates exhibited low micromolar efficacy against *C. albicans* , with MICs ranging from 15.62 to 31.25 μM for **Tat‐A** and 7.8–15.62 μM for **Tat‐B**, in contrast to the nonconjugated derivatives, which had MICs above 100 μM. **TAT** exhibited no activity against *C. albicans* , even at a concentration of 100 μM. The enhanced cationic characteristics due to **TAT** conjugation, along with the hydrophobic properties of the peptide aldehydes, facilitate improved permeation of the conjugates across fungal membranes, hence increasing their efficacy against fungal cells. It was indicated clearly that the interplay of chain length, hydrophobicity, and cationic characteristics is factors that affect the antifungal efficacy of peptides (Jiang et al. 2008; Margareta et al. 2002; De Zoysa et al. 2018). The conjugates demonstrated fungal proteasome inhibition at the minimum inhibitory concentration (MIC) of 31.25 μM for **Tat‐A** and 15.62 μM for **Tat‐B**.

Synergy testing by checkerboard assay was employed to evaluate the combined effect of **Tat‐A** and **Tat‐B** with the conventional antifungal agent AmB on *C. albicans* . The MIC values of **Tat‐A** and **Tat‐B** in conjunction with AmB were fivefold lower than when these peptides were evaluated independently. The MIC value of AmB in conjunction with **Tat‐A** and **Tat‐B** was reduced sixfold compared to AmB administered alone. FICI values of 0.36 and 0.35 were recorded for the AmB combination with **Tat‐A** and **Tat‐B**, respectively, both of which fall under the synergistic category limit. Prior research on antifungal lipopeptides demonstrated that AmB has hemolytic activity at 1.56 μM and is nonhemolytic at 0.25 μM (De Zoysa et al. 2018). The combination of Amb with conjugated peptide aldehydes exhibits antifungal activity at concentrations markedly lower than the hemolytic concentration of Amb, which is promising and suggests the potential for developing antifungal drug formulations that could address the existing toxicity concerns related to AmB.

Activity of Peptide Aldehydes on *Candida albicans* Biofilms: Addressing fungal biofilms and their advancement is arduous (Lohse et al. 2018). Biofilms in yeasts such as *C. albicans* evolve through stages of cell adhesion, germination, budding, filamentation, monolayer formation, proliferation, and culminate in full biofilm formation, an event that spans 24–48 h, contingent upon the environmental circumstances encountered. The *C. albicans* strain SC5314 is extensively utilized to investigate biofilm formation characteristics and serves as an exemplary model for evaluating antifungal agents (Ramage et al. 2002). The effectiveness of **Tat** conjugates in inhibiting and eliminating *C. albicans* biofilms was assessed by measuring the Minimum Biofilm Inhibitory Concentration (MBIC) and Minimum Biofilm Eradication Concentration (MBEC) via a quantitative colorimetric assay employing 2,3‐bis(2‐methoxy‐4‐nitro‐5‐sulfophenyl)‐2H‐tetrazolium‐5‐carboxanilide (XTT) dye. The minimum biofilm inhibitory concentrations (MBIC) of **Tat‐A** and **Tat‐B** were 15.6 and 7.8 μM, respectively, aligning with their minimum inhibitory concentrations (MIC) values, thereby validating that these peptide aldehydes impede *C. albicans* biofilms at their MICs. Treatment of 24‐h preformed biofilms of *C. albicans* with **Tat‐A** and **Tat‐B** resulted in the eradication of over 50% of biofilm cells at concentrations of 125 and 62.5 μM, respectively. They subsequently examined the impact of the TAT‐conjugated peptide aldehydes on the cellular morphology of the fungal biofilms at 2 × MBEC using SEM, which distinctly revealed substantial pore development and destruction of hyphae. AmB only induced pore development in the fungal membranes and exerted no influence on the fungal hyphae. A significant quantity of debris from hyphal rupture was clearly observable in the **Tat‐B** treated photos.

#### 2.2.2 Coumarin + CYP51 Inhibitors

Coumarin is a natural plant phenolic molecule with pharmacological effects including anticoagulant, antioxidant, anti‐inflammatory, and antibacterial properties (Flores‐Morales et al. 2023). Recent research shows that coumarin has antibiofilm activity against *C. albicans* , reducing adherence and morphological transformation (Xu et al. 2019).

**FIGURE 6:**
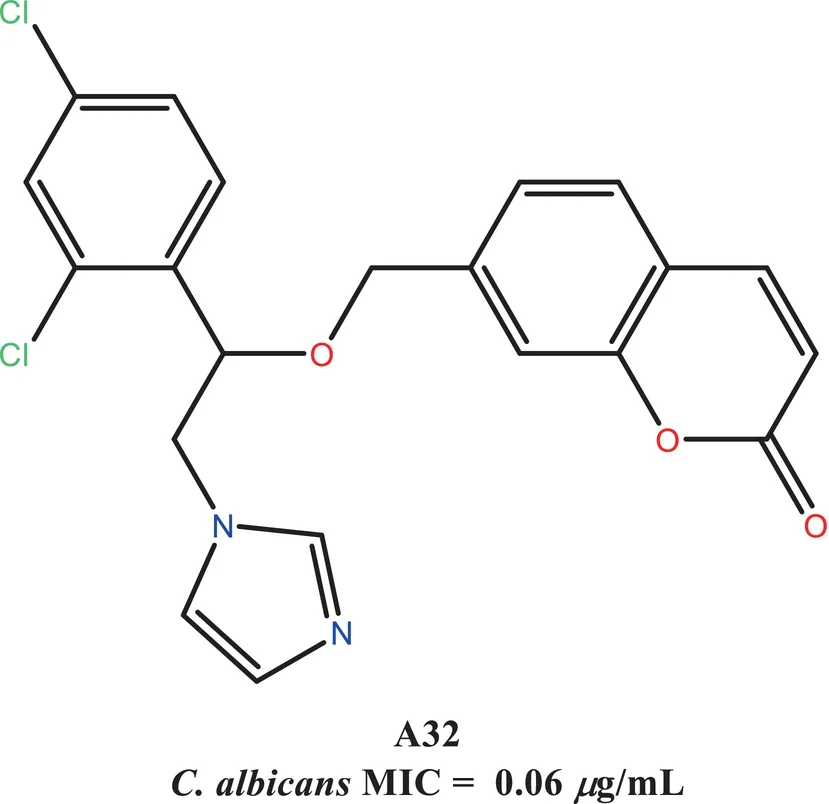
Hybrid of coumarin and traditional CYP51 inhibitor (azole).

This work combined coumarin, which possesses antibiofilm properties, with traditional CYP51 inhibitors to achieve antidrug resistance efficacy. The subsequent experimental results indicated that the target compound could inhibit the activity of both pathogenic and drug‐resistant fungi. Additionally, preliminary studies on the mechanism of action demonstrated that the target compound inhibited ergosterol biosynthesis by targeting lanosterol 14α‐demethylase (CYP51). Moreover, chemical **A32** (Figure 6) compromised the fungal cell wall and membrane, as evidenced by TEM. The resistance mechanisms indicated that target chemicals could diminish fungal pathogenicity by obstructing the yeast‐to‐hypha morphological transition and biofilm development, while concurrently downregulating genes associated with biofilm formation. Moreover, representative substances may mitigate drug resistance by downregulating the expression of four drug‐resistance genes (ERG11, CDR1, CDR2, and MDR1). Compound **A32** exhibited fungicidal properties against fluconazole‐resistant *C. albicans* (strain 904) and markedly increased intracellular ROS levels, leading to cellular damage. Moreover, compound **A32** shown efficacy in treating both fluconazole‐sensitive and fluconazole‐resistant fungal infections in vivo, dramatically diminishing kidney fungal burden. Additionally, compound **A32** exhibited favorable results in preliminary pharmacokinetic assessments and toxicity evaluations. The hybrid molecule **A32** is a viable possibility for the treatment of resistant candidiasis (Yan et al. 2023).

#### 2.2.3 CYP51 + Hsp90 Inhibitors

Hsp90, a crucial regulator of fungal pathogenicity in *C. albicans* , is a potential antifungal target. It regulates stress adaptation, yeast‐to‐hypha morphological transition, and biofilm formation (Shapiro et al. 2009; O'Meara et al. 2017; Taipale et al. 2010).

**FIGURE 7:**
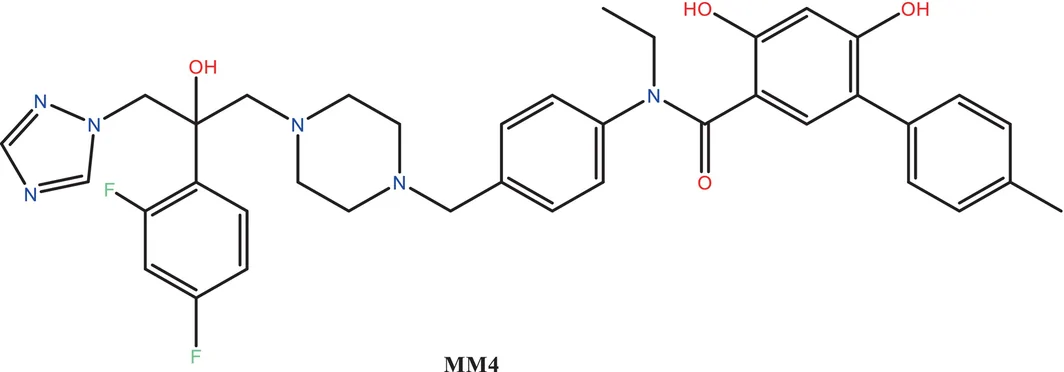
Dual inhibitor of CYP51 and Hsp90.

The initial generation of CYP51/Hsp90 dual inhibitors was developed and manufactured for the treatment of IC. Specifically, compound **MM4** (Figure 7) significantly suppressed the proliferation of *C. albicans* SC5314 cells. Compound **MM4**, a dual inhibitor of CYP51 and Hsp90, obstructed the ergosterol production pathway, showed strong binding affinity to fungal Hsp90, and exhibited remarkable inhibitory efficacy against the hsp90Δ/HSP90 *C. albicans* SC5314 mutant. Furthermore, compound **MM4** had significant inhibitory effects on both filamentation and biofilm development. Notably, compound **MM4** markedly decreased the renal load of *C. albicans* SC5314 in murine subjects. The identification of CYP51/Hsp90 dual inhibitors offers a novel therapeutic approach for IC. Ongoing structural optimization and examination of the antifungal mechanisms of the CYP51/Hsp90 dual inhibitors are underway (Zhang et al. 2025).

### 2.3 Antifungal + Host Immunity Modulation

#### 2.3.1 CYP51 + HDAC Inhibitors

Lanosterol 14α‐demethylase (CYP51) is the target of azole antifungal drugs, including fluconazole (FLC), itraconazole (ITC), and voriconazole (VOR). Azoles decrease the formation of ergosterol in fungal cell membranes by targeting CYP51, hence exhibiting fungistatic action (Liu et al. 2014). The primary molecular pathways of azole resistance encompass the overexpression of CYP51, structural alterations in CYP51, activation of efflux pumps, and the development of fungal biofilm. Histone deacetylases (HDACs), a family of epigenetic enzymes, are prevalent in eukaryotic cells.

Recent findings indicate that fungal HDACs significantly contribute to the emergence of drug resistance, particularly to azoles (Robbins et al. 2012; Lamar and Edlind 2002; Kmetzsch 2015). Moreover, certain fungal biological characteristics and pathogenicity are functionally modulated by HDACs (Lamoth et al. 2015; Kim et al. 2015; Garnaud et al. 2016).

**FIGURE 8:**
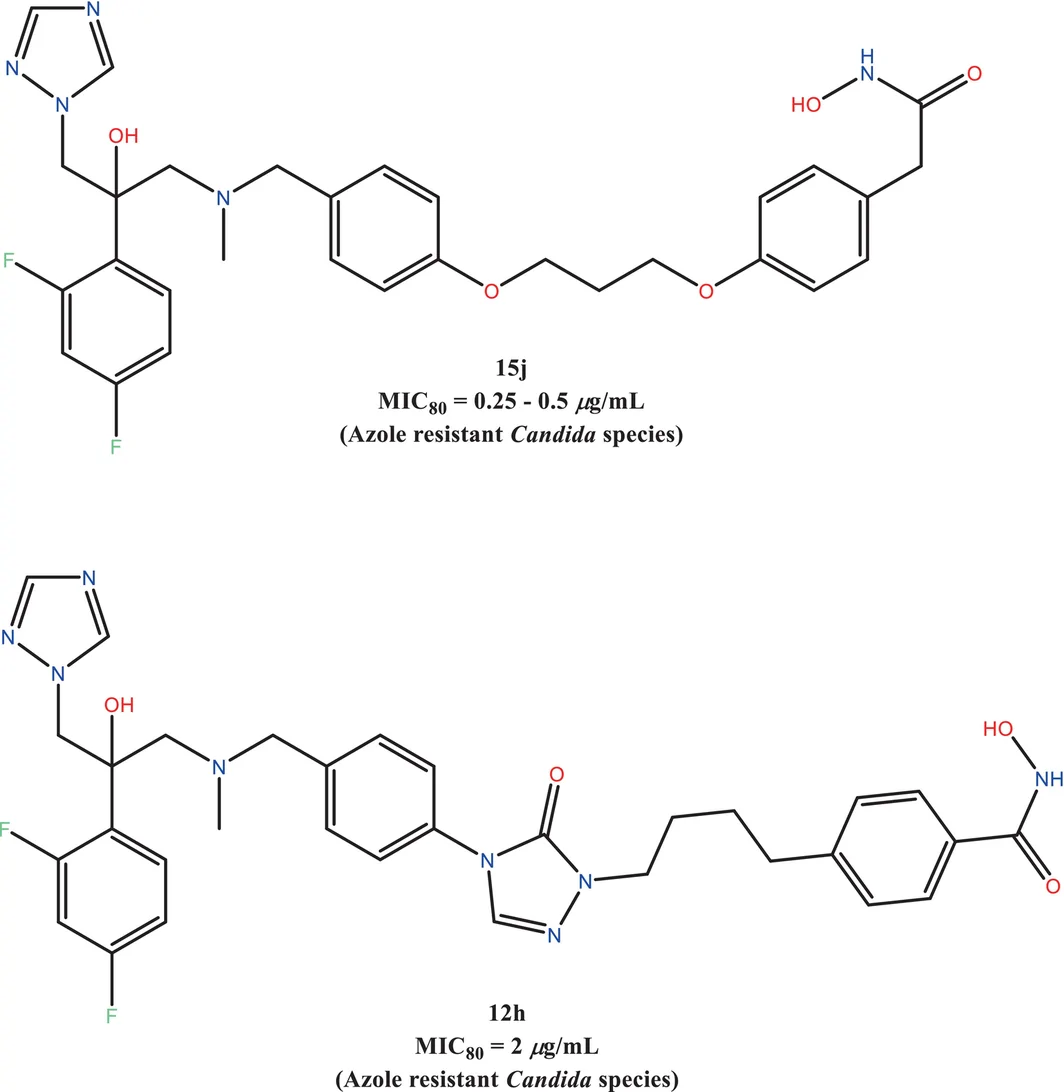
Dual inhibitors targeting both lanosterol 14α‐demethylase (CYP51) and histone deacetylase (HDAC).

Another study by C. Sheng and group presents the design of the inaugural generation of dual inhibitors targeting lanosterol 14α‐demethylase (CYP51) and histone deacetylase (HDAC), demonstrating significant antifungal efficacy against azole‐resistant clinical isolates. Specifically, compounds **12h** and **15j** (Figure 8) showed significant activity both in vitro and in vivo against azole‐resistant candidiasis. Studies on antifungal mechanisms demonstrated that they inhibit ergosterol biosynthesis and HDAC catalytic activity in fungi, hence reducing efflux pump function, yeast‐to‐hypha morphological transition, and biofilm development. CYP51‐HDAC dual inhibitors constitute a viable approach for the development of new antifungal drugs targeting azole‐resistant candidiasis (Han et al. 2020).

Development of dual inhibitors targeting CYP51 and HDAC: In prior research, a comprehensive structure‐based design and structure–activity relationship (SAR) analysis of azole CYP51 inhibitors was conducted and a series of HDAC‐based multitargeting inhibitors was developed, establishing a foundation for the rational design of CYP51/HDAC dual inhibitors. The design of the CYP51/HDAC dual inhibitors involved the integration of the essential pharmacophores characteristic of both CYP51 and HDAC inhibitors.

Typically, the classical HDAC inhibitor, such as SAHA, comprises a surface recognition cap (SRC) group, a linker, and a zinc‐binding group (ZBG), which may include hydroxamic acid. A linker of specific length and ZBG are recognized as essential pharmacophores of HDAC inhibitors, whereas various scaffolds may be devised as SRC groups (Chen et al. 2019; Zhang et al. 2019). The principal pharmacophores of conventional CYP51 inhibitors are characterized by a triazole ring, which coordinates with the heme iron atom of CYP51, and a difluorophenyl (or dichlorophenyl) ring that interacts with the hydrophobic pocket of CYP51. Additionally, various side chains of differing lengths can be accommodated within the active site of CYP51 (Che et al. 2009). Recently, the crystal structure of *Candida albicans* CYP51 in complex with posaconazole has been elucidated. The binding mode of posaconazole suggests a notable correlation between the length of azoles and their inhibitory activity against CYP51, with longer side chains potentially facilitating further interactions within the substrate access channel of CYP51. Based on the preceding analysis, the type A CYP51/HDAC dual inhibitors were conceived by integrating a linker and hydroxamic acid ZBG into the structure of FLC. Subsequently, a range of *N*‐methylbenzylamine containing linkers were examined drawing upon our prior insights in the development of CYP51 inhibitors (Che et al. 2009). In order to substantiate the design rationale, compound **15j** was chosen for docking into the active site of CYP51. The findings indicated that it exhibited a comparable binding mode to posaconazole, with the ZBG and linker group positioned within the channel of CYP51.

#### 2.3.2 NLRP3 Inflammasome + AHAS inhibitor

In the last ten years, an increasing amount of literature has clarified the significant function of the NLRP3 inflammasome in orchestrating innate inflammatory responses to pathogenic fungus, particularly the *Candida* species (Camilli et al. 2020). Inflammasomes are a group of mammalian cytosolic receptors that, upon detecting endogenous or exogenous DAMPs, undergo conformational alterations to activate Caspase‐1, which cleaves and releases mature interleukin‐1 beta (IL‐1β) to facilitate innate immune signaling and neutrophil recruitment. Previously thought to be exclusive to hematopoietic cells, new research has demonstrated that inflammasomes, including the NLRP3 type, are functionally present in other cell types, such as epithelial cells (Thinwa et al. 2014; Bui et al. 2016; Wu et al. 2020).

Consequently, if meticulously regulated, the NLRP3 inflammasome could serve as a promising target to mitigate immune responses and avert uncontrolled inflammatory diseases during fungal infections. Nevertheless, its suppression may also diminish fungal recognition and initial containment; therefore concurrent control of fungal proliferation would be advantageous.

AHAS catalyzes the condensation of two pyruvate molecules to produce acetolactate, which is utilized in the synthesis of the branched chain amino acids valine or leucine. Alternatively, pyruvate can react with 2‐ketobutyrate to yield acetohydroxybutyrate, which is then used to synthesize isoleucine (Liu, Li, and Wang 2016). Inhibition of AHAS leads to stunted growth in conditions where branched chain amino acids cannot be imported or produced effectively from precursor molecules (McCourt and Duggleby 2006).

**FIGURE 9:**
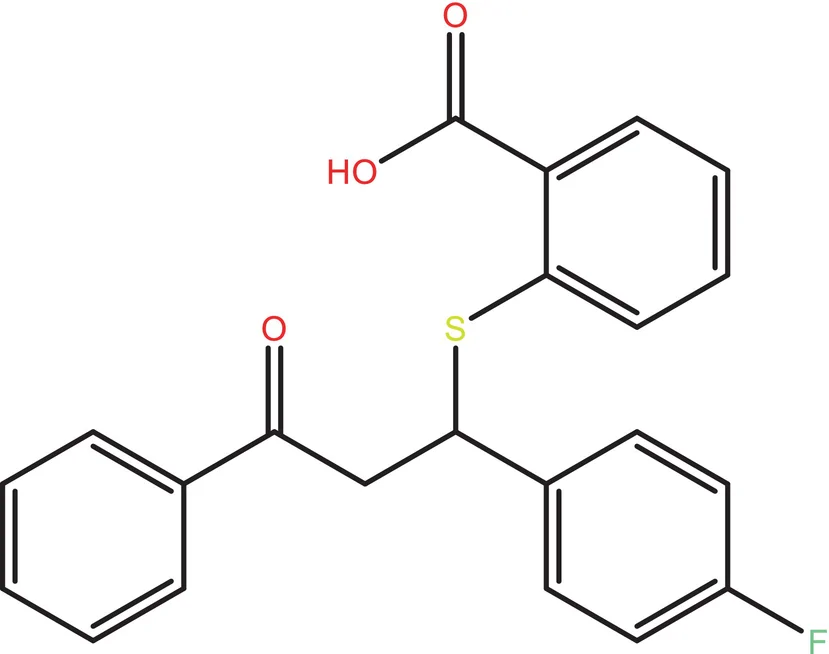
Dual Inhibitors of the NLRP3 inflammasome and fungal acetohydroxyacid synthase (AHAS).

There are notable structural parallels between the well‐characterized inhibitors of the NLRP3 inflammasome and those targeting fungal acetohydroxyacid synthase (AHAS). Consequently, K. E. Hevener and B. M. Peters and their colleague utilized this information to perform an in silico molecular docking screen aimed at discovering novel polypharmacologic inhibitors of these targets, which led to the identification of 12 candidate molecules. Among these, compound **10** (Figure 9) markedly reduced the activation of the NLRP3 inflammasome induced by LPS + ATP, while also exhibiting growth inhibitory effects against *C. albicans* , which were diminished in the presence of exogenous branched chain amino acids, aligning with the targeting of fungal AHAS. SAR investigations have elucidated a crucial molecular framework necessary for dual functionality. In conclusion, both **10** and its analog **10a** demonstrated low micromolar potency, yielding low IC_50_ values for IL‐1β release and MIC_50_ values for fungal growth against various *Candida* species. This body of work collectively illustrated the promising potential inherent in dual‐target methodologies for the enhanced management of fungal infections (Lowes et al. 2021).

#### 2.3.3 COX + CYP51 Inhibitors

**FIGURE 10:**
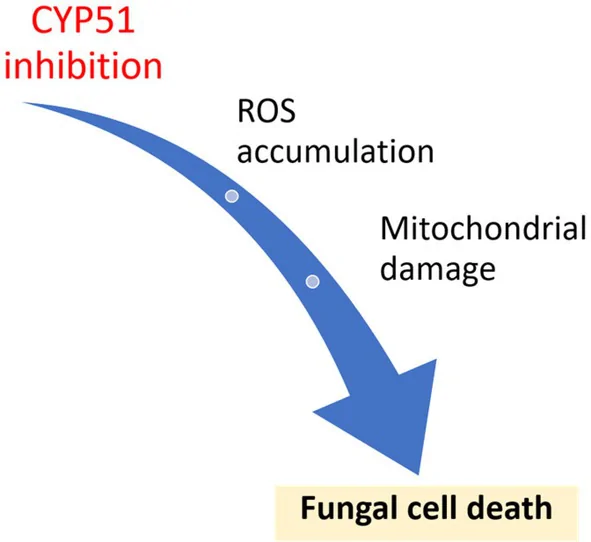
Common mechanistic pathway adopted by COX + CYP51 dual inhibitors against *Candida albicans* .

The arachidonic acid route is linked to yeast‐to‐hyphae morphogenesis in various Candida species (Calderone and Fonzi 2001; Lee et al. 2021). Prostaglandins participate in the morphogenesis and pathogenicity of yeast and facilitate the host's inflammatory response (Erb‐Downward and Noverr 2007; Alem and Douglas 2004). Prostaglandin E2 (PGE2) modulates growth and colonization while facilitating the development of biofilms in certain *Candida* species (Mishra et al. 2014; Petra and Nigel 2017). Numerous studies indicate that diminished PGE2 synthesis restricts the virulence of pathogenic fungi, implying that employing inhibitors of the arachidonic acid pathway may enhance the prognosis of fungal infections (Tan et al. 2019; Liu, Wang, et al. 2016; Noverr et al. 2001). These dual ligands commonly follow the mechanism as shown in Figure 10.

It has been previously proven that nonsteroidal COX‐inhibiting anti‐inflammatory medications and azole antifungals exhibit synergy to enhance antifungal efficacy (Alem and Douglas 2004; Liu, Wang, et al. 2016; Pina‐Vaz et al. 2000; Scott et al. 1995; de Quadros et al. 2011; Urai et al. 2014; Arai et al. 2005). Combination therapy may be influenced by variations in pharmacological characteristics and by the adverse effects of the drug combination. To address potential difficulties associated with combinations of azole antifungals and COX inhibitors, R. Elias and her colleagues synthesized a new antifungal by conjugating an azole pharmacophore with a COX inhibitor to create a hybrid medicinal molecule. These hybrids were synthesized by conjugating a chiral azole pharmacophore with a series of chiral and achiral COX inhibitors, resulting in the formation of 24 chiral hybrids.

The antifungal activity profiles of the hybrids were evaluated against a comprehensive panel of Candida species, encompassing seven of the most prevalent species of this common fungal infection, and compared to the efficacy of the therapeutically utilized azole medications FLC and VOR. The antifungal efficacy of many hybrids surpassed that of FLC. Two hybrids, ibuprofen‐based **1** and flurbiprofen‐based **5**, distinguished themselves due to their potency, which was much greater than FLC and comparable to VOR.

Analysis of the structure–activity relationship demonstrated that all hybrids featuring an S‐configured azole pharmacophore exhibited greater antifungal potency compared to their R‐configured counterparts. No generalization can be applied to the chiral COX inhibitors. In all hybrids including a chiral COX inhibitor, the influence of the chiral center of the azole pharmacophore on the antifungal activity of the hybrids was significantly greater than that of the chiral center of the COX inhibitor.

Significantly, the examination of tolerance, characterized as the capacity of a subpopulation of cells to proliferate in the presence of the medication, indicated that yeast cultures exhibited reduced tolerance when exposed to hybrids **1** and **5** compared to FLC and VOR. Clinical isolates with elevated tolerance correlate with persistent infections, indicating that diminished medication tolerance may decrease the likelihood of infection persistence and/or recurrence.

**FIGURE 11:**
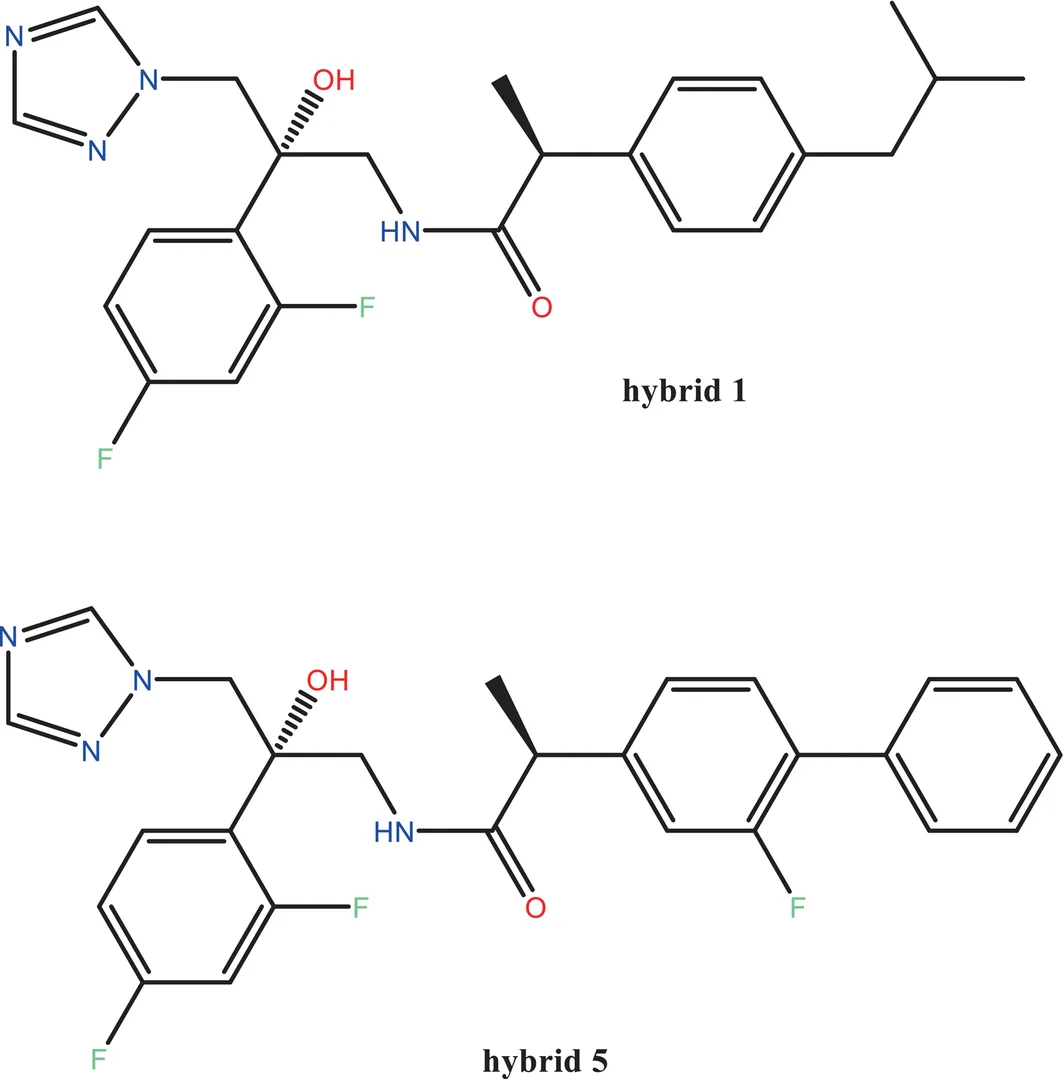
Dual‐acting hybrids capable of inhibition of CYP51 and COX.

Mechanistic analysis demonstrated that, in contrast to the therapeutically utilized FLC and VOR, which primarily target CYP51, hybrids **1** and **5** (Figure 11) maintained efficacy against an erg3ΔΔ/erg11ΔΔ mutant *C. albicans* strain that is deficient in CYP51. This activity was markedly inferior to that of these hybrids against the parent *C. albicans* strain from which the target‐lacking mutant was produced. This suggests that the antifungal efficacy of these dual‐acting hybrids primarily stems from the inhibition of CYP51; however, in contrast to FLC and VOR, the hybrids also exert a secondary mechanism of action attributed to the COX‐inhibiting component (Elias et al. 2022).

This work provides directions for the creation of effective antifungal medicines that combine the antifungal properties of azole antifungals and COX inhibitors in hybrid molecules.

These novel antifungals have significant antifungal efficacy and, crucially, decreased tolerance levels. The dual‐acting hybrids presented herein provide promising candidates for subsequent clinical advancement.

**FIGURE 12:**
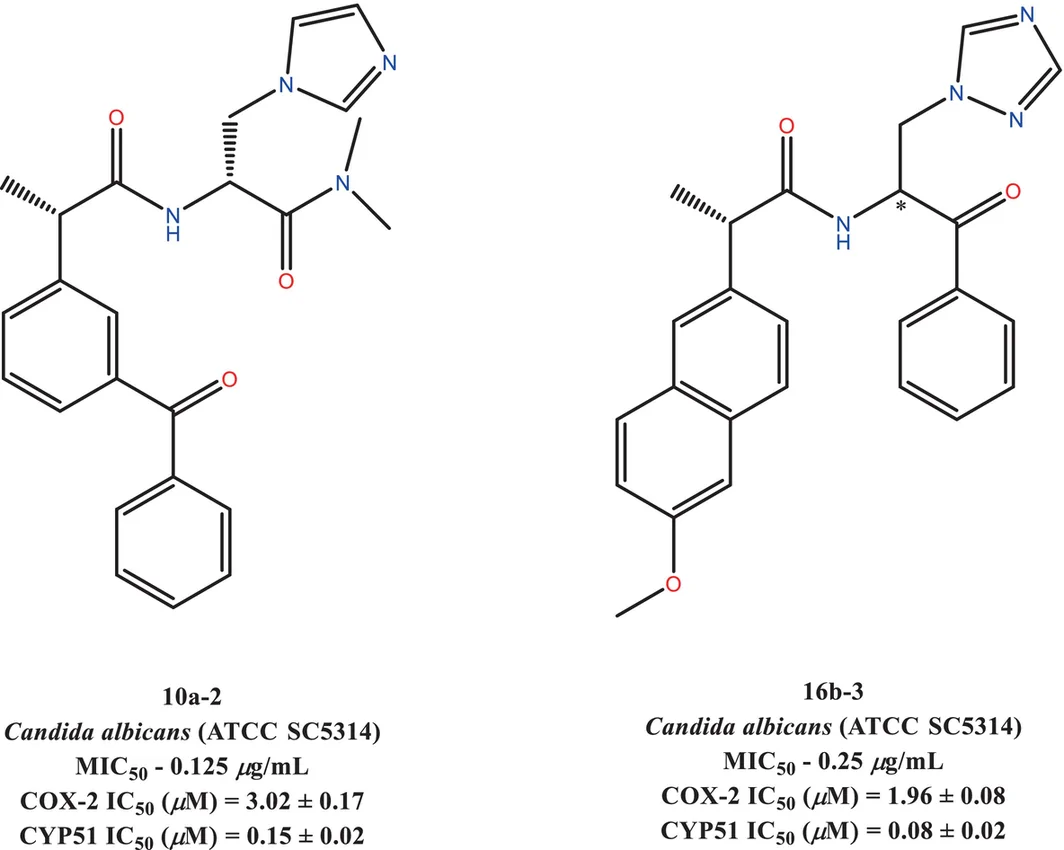
Dual‐acting inhibitors of CYP51 and COX‐2.

Bin Sun and his team developed three distinct series of novel compounds through skeleton screening and combination, based on a systematic analysis of the binding mode of the COX‐2 inhibitor and CYP51 inhibitor. Several small molecules were synthesized and characterized. Subsequently, their antifungal and anti‐drug‐resistant fungal properties were assessed. Among them, the compounds **10a‐2** and **16b‐3** (Figure 12) were chosen for subsequent mechanistic investigations due to their exceptional biological activity (MIC_50_, 0.125–2.50 μg/mL) in vivo and in vitro. On the one hand, the compounds **10a‐2** and **16b‐3** have the potential to inhibit CYP51 (IC_50_, 0.08 ± 0.02–0.15 ± 0.02 μM) and disrupt the fluidity and permeability of the fungal cell membrane in vitro, thereby blocking the ergosterol synthesis pathway (Liu, Liu, Fan, et al. 2022).

The Mitochondrial membrane polarization (ΔΨm) decrease further induced the rapid accumulation of ROS and mitochondrial damage, which ultimately resulted in the apoptosis phenomenon. Conversely, the target compounds (**10a‐2, 16b‐3**) also effectively inhibited COX‐2 activity (IC_50_, 1.96 ± 0.08–3.02 ± 0.17 μM), eliminated the inflammatory reaction of the infected region by reducing the expression of the inflammation‐inducing enzyme (COX‐2, MMP‐9, NLRP3), and activated the body's immune function by increasing CD3+ and CD8+ T cell levels. This further expedited the rehabilitation process of fungal infection. In conclusion, this study has introduced a novel approach and strategy for the development of inhibitors that can simultaneously exhibit antifungal and anti‐inflammatory properties in vitro and in vivo.

**FIGURE 13:**
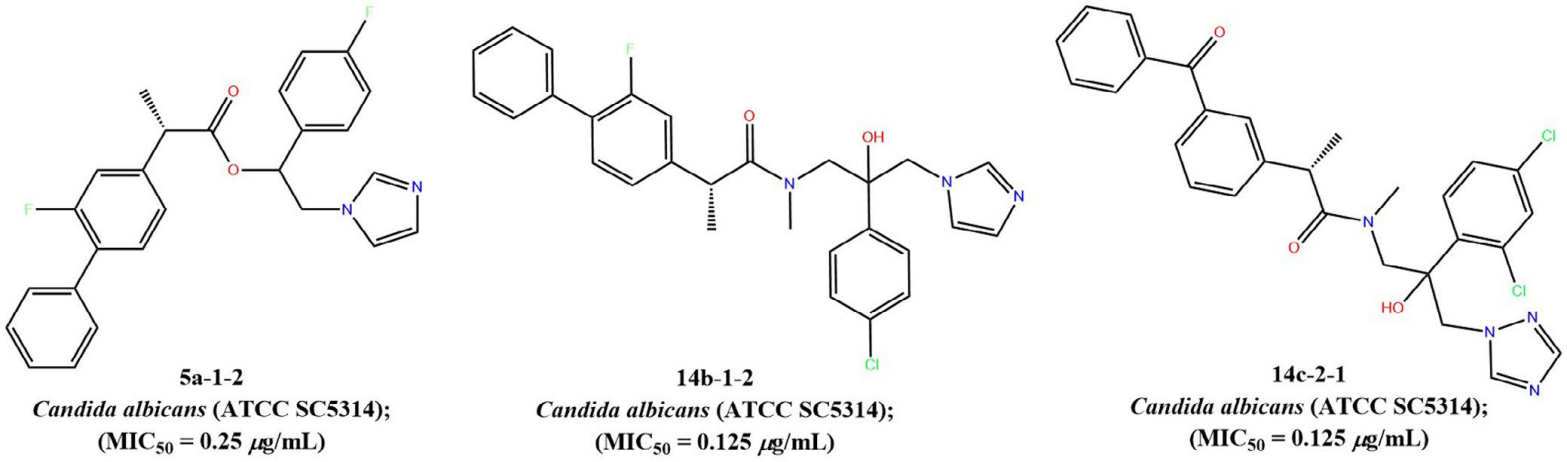
Dual‐acting inhibitors of CYP51 and COX‐2.

Apart from the previous work, this work also offered an approach for constructing dual‐target inhibitors. Skeleton screening and structural splicing were conducted following a comprehensive investigation of the dual‐target enzyme, resulting in the design and synthesis of three distinct series of new compounds. The majority of the compounds demonstrated adequate antifungal efficacy. Compounds **5a‐1‐2, 14b‐1‐2**, and **14c‐2‐1** (Figure 13) showed exceptional biological activity against a wide range of pathogenic fungi, leading to the selection of drug‐resistant fungi for the ensuing mechanistic investigation. These chemicals may impede CYP51 enzyme activity and disrupt the osmotic pressure and fluidity of the fungal cell membrane. This efficiently encouraged the formation of reactive oxygen species in fungal cells by generating mitochondrial damage, resulting in the occurrence of fungal death. Conversely, these drugs suppressed COX‐2 enzyme activity, diminished the expression of inflammation‐inducing proteins, and stimulated the immune system. This expedited the restoration of the fungus‐infected area. Consequently, these compounds rapidly attained synergistic antifungal and anti‐inflammatory therapeutic properties (Liu, Liu, Yu, et al. 2022).

#### 2.3.4 CYP51 + PD‐L1 Inhibitors

Fungal infections, including drug‐resistant fungi, are challenging to eliminate because of the immunological escape of fungal cells in the infection area (Lionakis et al. 2023; Jenks et al. 2020). Exotoxin secreted by pathogenic fungi can cause increased activation of PD‐L1 in the infection site (Spec et al. 2016; Yu et al. 2022).

This can weaken the immune system and help dangerous fungus escape (Wurster, Watowich, and Kontoyiannis 2022).

**FIGURE 14:**
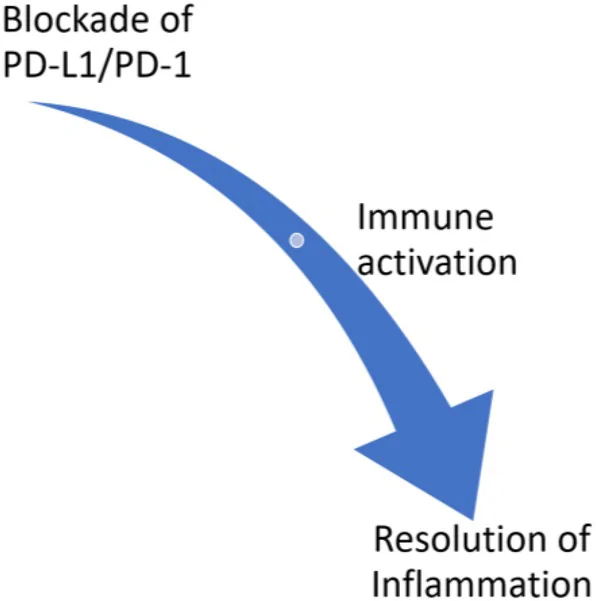
Commonly adopted mechanistic pathway for the PD‐L1/PD‐1 inhibitors.

Currently, PD‐1/PD‐L1 drugs have been shown to treat fungal infections by blocking human immune checkpoints (Figure 14; Wurster, Albert, Bharadwaj, et al. 2022). In combination with CYP51 inhibitors, the body's recovery process for pathogenic or drug‐resistant fungal infections accelerated (Yang et al. 2020; Wurster, Albert, and Kontoyiannis 2022).

**FIGURE 15:**
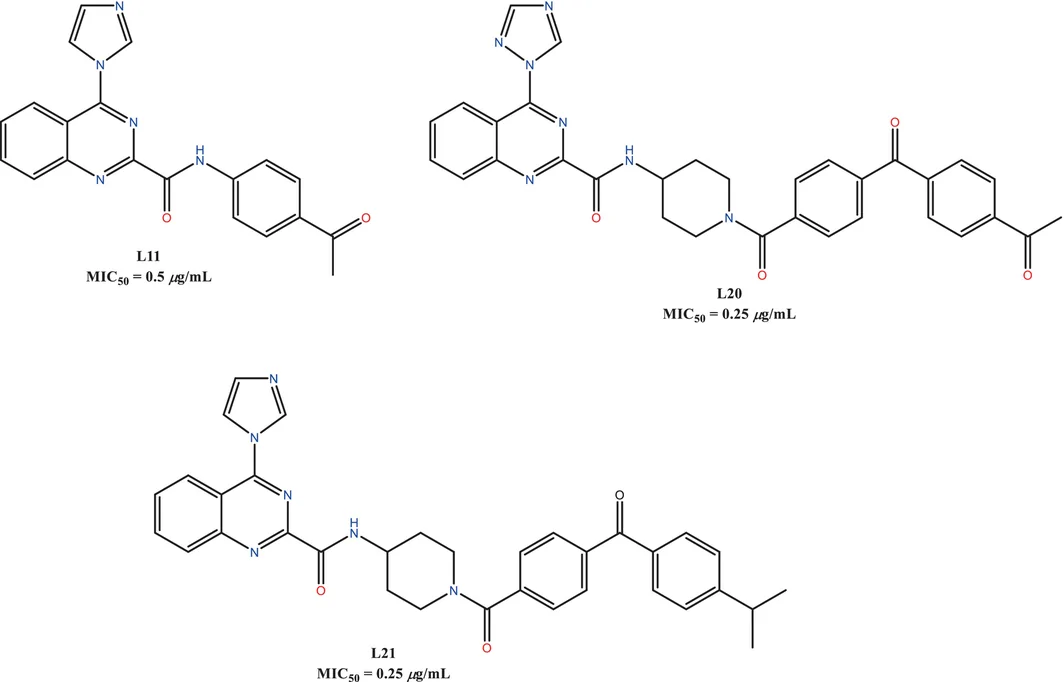
Inhibitors capable of dual‐targeting of CYP51 and PD‐L1.

The dual‐target (CYP51/PD‐L1) is significant in fungal proliferation and immune suppression. A series of new quinazoline compounds exhibiting dual‐target inhibition was developed utilizing the skeleton growth approach, followed by synthesis, characterization, and evaluation of their structures. The optimized compounds (**L11**, **L20**, **L21**) (Figure 15) were chosen for additional investigation due to their significant biological activity against several fungal strains (MIC_50_, 0.25–2.0 μg/mL) in vitro. These chemicals reduced CYP51 activity, produced ROS accumulation, and caused mitochondrial damage, ultimately resulting in fungal lysis and death. Conversely, they also successfully stimulated the body's immune response by inhibiting the connection between PD‐L1 and PD‐1, mitigated the inflammatory response, and expedited the recovery from fungal infections (Sun et al. 2023).

**FIGURE 16:**
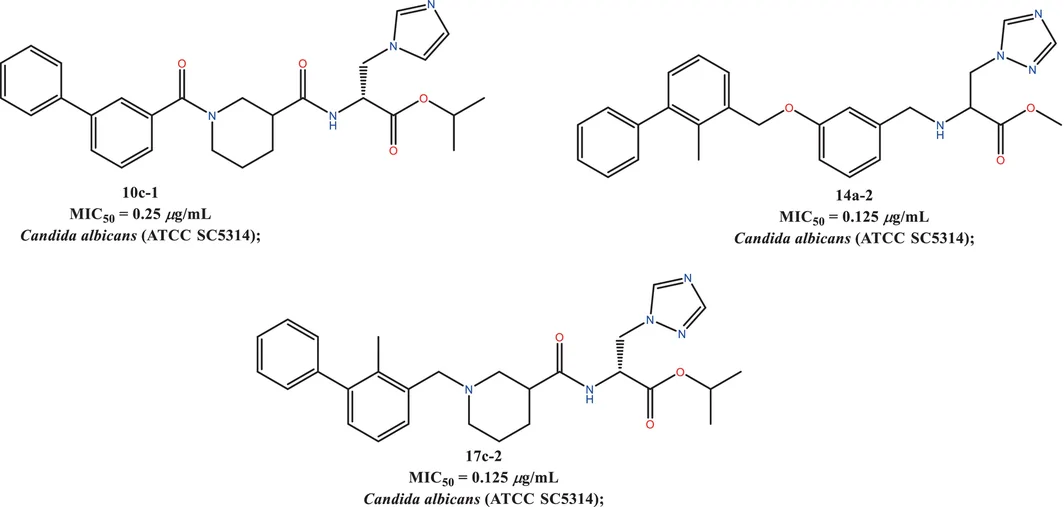
Inhibitors for dual‐targeting of CYP51 and PD‐L1.

Pathogenic fungi can intensify the degree of degeneration by increasing their reproduction and suppressing the body's immunological function. As mentioned earlier, PD‐L1 and CYP51, as pivotal dual‐target enzymes, significantly contribute to the progression. Following the aforementioned analysis, several series of novel dual‐target compounds were devised by the approach of fragment combination, and these molecular structures were subsequently synthesized, described, and assessed for activity. The target compounds (**10c‐1, 14a‐2, 17c‐2**) (Figure 16) showed remarkable antifungal and antidrug‐resistant fungal efficacy in vitro. The favored chemical **14a‐2**, exhibiting high‐efficiency dual‐target inhibition, can impede fungal proliferation, induce mitochondrial impairment, and finally result in fungal cell lysis and death. To further validate the therapeutic capabilities of these compounds, the appropriate COF (Bt‐Bch) carrier was successfully synthesized to enhance their bioavailability, demonstrating significant targeted delivery capability. These chemicals not only successfully suppressed the proliferation of drug‐resistant fungi in vivo, but also stimulated the body's immune response by obstructing the connection between PD‐L1 and PD‐1. This subsequently diminished the body's inflammatory reaction and expedited the recovery process from the fungal infection. This study advanced the scientific domain of antifungal drug development and presented a viable potential strategy for the clinical management of fungal diseases (Liu et al. 2023).

**FIGURE 17:**
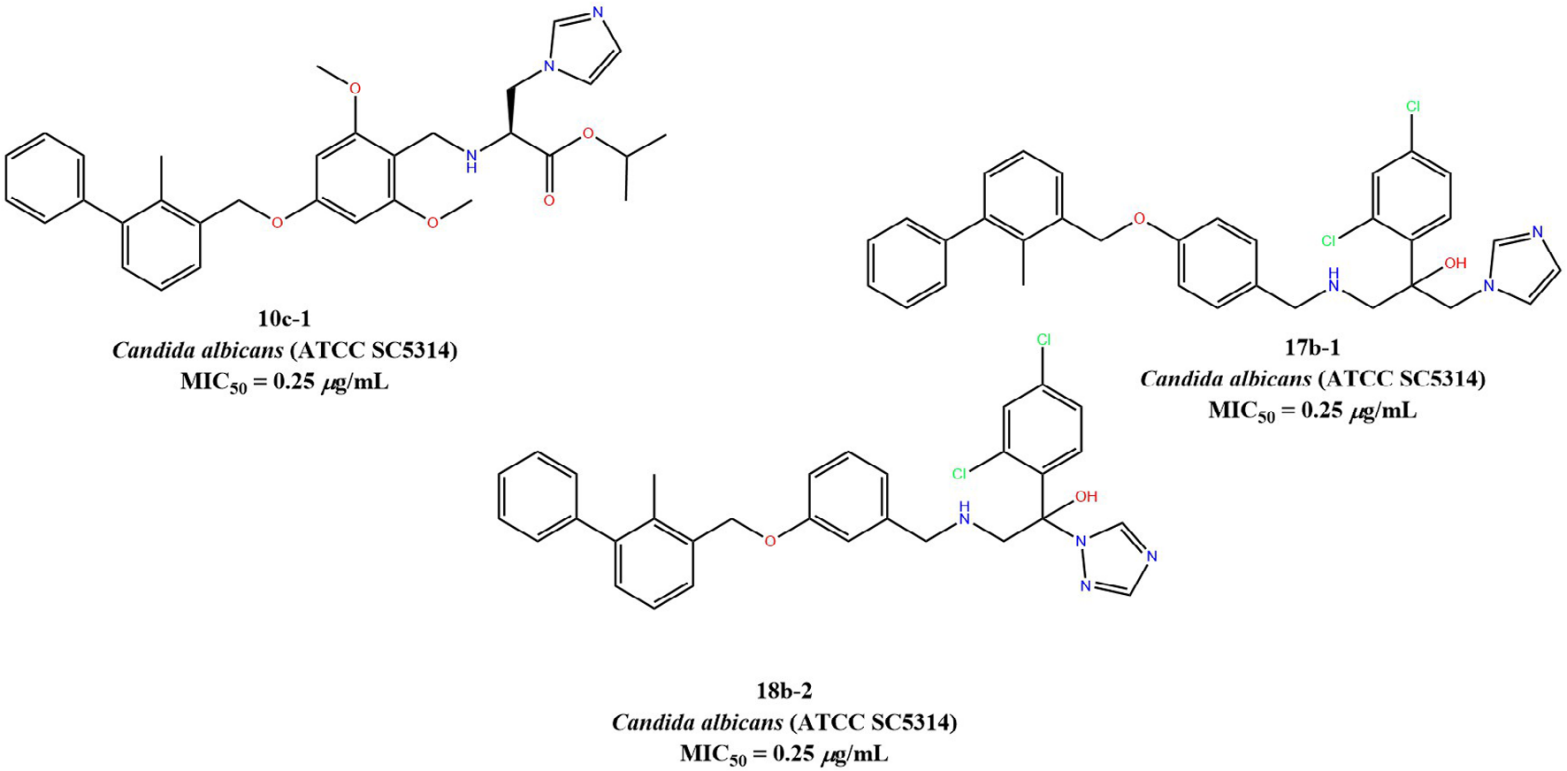
Inhibitors capable of dual‐targeting of CYP51 and PD‐L1.

The dual‐target (PD‐L1/CYP51) drug design technique was developed to facilitate the development of novel compounds; their molecular structures were designed, synthesized, characterized, and assessed by Sun and his colleagues. The majority of the compounds exhibited suitable antifungal efficacy. The selected compounds (**10c‐1, 17b‐1, and 18b‐2**) (Figure 17) showed effective antifungal and antidrug‐resistant fungal activity in vitro. Subsequently, their mechanism of action was further investigated. These chosen chemicals may obstruct the biosynthesis process of fungal ergosterol by inhibiting CYP51, resulting in an accelerated increase in ROS levels, aggravating mitochondrial damage, and ultimately causing the lysis and demise of fungal cells. Conversely, these chemicals additionally enhanced the body's immune response by obstructing the PD‐L1/PD‐1 connection and expedited the resolution of inflammation, hence achieving excellent treatment efficacy for fungal infections. Additionally, the appropriate COF (Tp‐Bch) carrier was successfully synthesized, enabling the targeted delivery of the chosen chemical **18b‐2** to the infection site and enhancing its bioavailability. This work not only advanced the development of antifungal medications but also identified viable candidate chemicals and carriers for the clinical treatment of fungal infections (Yu et al. 2024).

The dual‐target (CYP51/PD‐L1) receptor is integral to the mechanisms of fungal infection, influencing both fungal proliferation and the immunological response of the host. Consequently, this study presented a dual‐target inhibition method. Three distinct series of new compounds were developed utilizing the skeleton splicing technique, informed by dual‐target characteristics. Their chemical structures were synthesized and described, followed by an evaluation of their corresponding activity.

**FIGURE 18:**
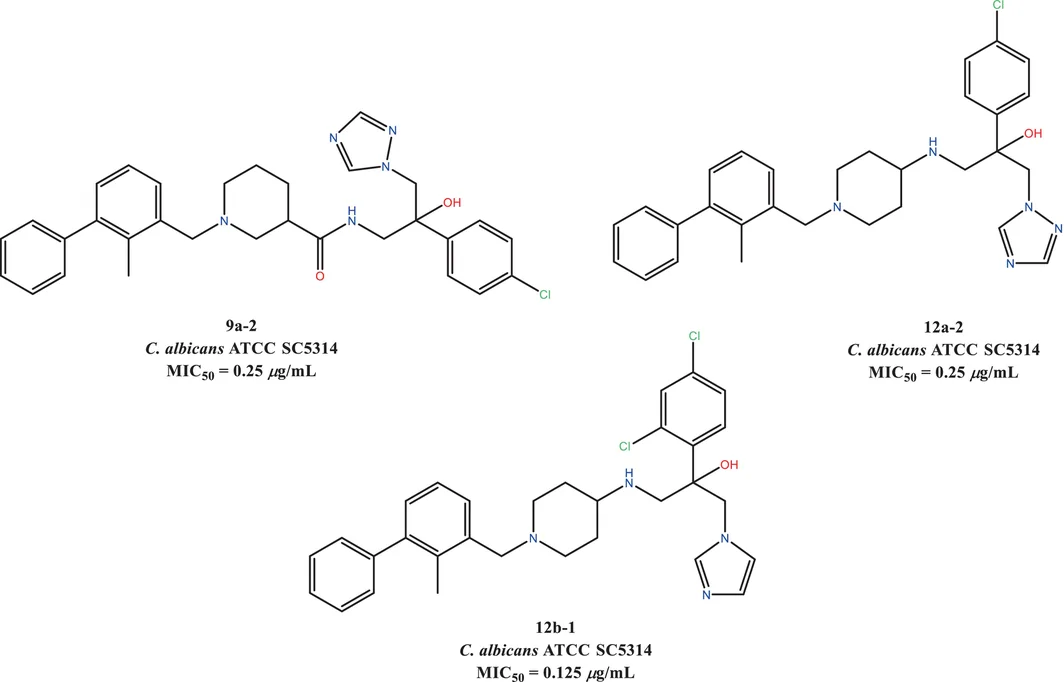
Inhibitors for dual‐targeting of CYP51 and PD‐L1.

Among the novel compounds, **9a‐2, 12a‐2**, and **12b‐1** (Figure 18), with MIC_50_ values ranging from 0.125 to 2.0 μg/mL, demonstrated exceptional antifungal and antidrug‐resistant fungal activity in vitro, particularly compound **12a‐2**, which exhibited the most extensive spectrum of biological activity against various pathogenic fungal strains. Concurrently, the mechanism of action was examined, revealing that these drugs can both limit fungal proliferation and activate the immune system by targeting dual pathways (CYP51/PD‐L1). Consequently, the nanocomposites (**Nc‐12a‐2**) were then developed utilizing LDH as a vehicle for compound **12a‐2**, enabling precise delivery of the target compound **12a‐2** to the infection site. Consequently, their pharmacological effects were assessed in vivo. The research validated that the favored compound **12a‐2**, derived from **Nc‐12a‐2**, demonstrated highly effective biological activity in addressing both deep and superficial fungal infections, facilitating the body's inflammation resolution and resulting in effective treatment of fungal infections (Liu, Yu, et al. 2024).

### 2.4 Azole Sensitizers

#### 2.4.1 JAK2 + HDAC Inhibitors

The Janus kinases (JAK1, JAK2, JAK3, and TYK2) are part of the intracellular protein tyrosine kinase family, which are crucial for the signaling of many cytokines (Laurence et al. 2012). The activation of JAKs by various cytokines leads to the phosphorylation and dimerization of STAT (signal transducers and activators of transcription) proteins, which subsequently translocate to the nucleus to initiate gene transcription (O'Shea and Plenge 2012). The JAK/STAT signaling pathway is linked to various biological functions, including inflammation, immunological response, and hematopoiesis (Shuai and Liu 2003; Clark et al. 2014).

Histone deacetylases (HDACs), a class of epigenetic enzymes, remove acetyl groups from lysine residues on histones and other proteins, significantly influencing many physiological activities such as gene regulation, transcription, and cell proliferation, differentiation, and apoptosis. These are crucial modulators of heat shock protein 90 (Hsp90), which is vital for fungal viability and linked to antifungal medication resistance (Tiwari et al. 2015). HDAC inhibitors were documented to augment the efficacy of antifungal drugs (e.g., azoles and echinocandins) against diverse fungal infections, including resistant strains (Lamar and Edlind 2002; Li et al. 2015; Mai et al. 2007).

**FIGURE 19:**
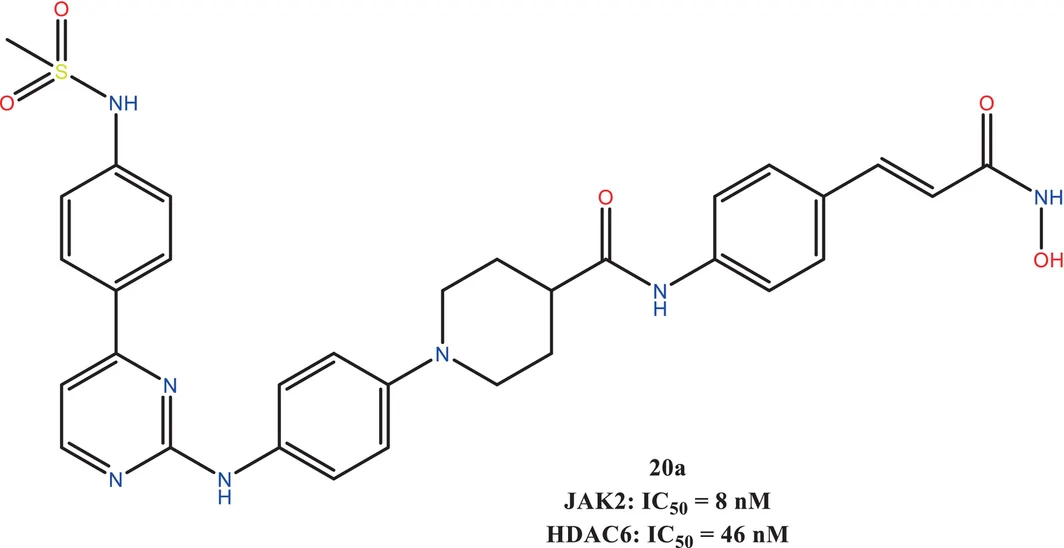
A potent and selective dual inhibitor of JAK2 and HDAC6.

Leukemia patients frequently experience the diminished effectiveness of treatment and elevated susceptibility to infections caused by invasive fungal pathogens. A novel therapeutic technique was presented by a small molecule that can concurrently address leukemia and invasive fungal infections (IFIs). Newly found dual inhibitors of Janus kinase 2 (JAK2) and histone deacetylase (HDAC) exhibit significant antiproliferative effects on hematological cell lines and demonstrate remarkable synergistic efficacy with fluconazole in the treatment of resistant *Candida albicans* infections. Compound **20a** (Figure 19), a potent and selective dual inhibitor of JAK2 and HDAC6, had remarkable in vivo antitumor activity in various acute myeloid leukemia (AML) models and shown synergy with fluconazole in treating resistant *C. albicans* infections (Huang et al. 2018).

**FIGURE 20:**
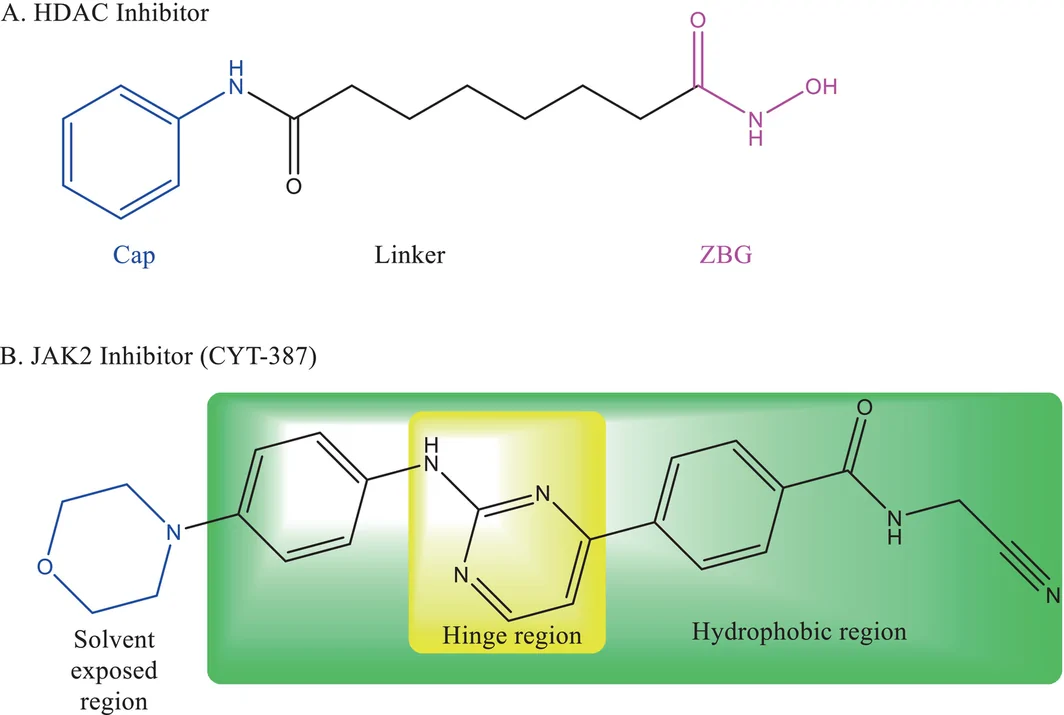
Structure of selective HDAC inhibitor (above) and selective JAK2 inhibitor (below), which inspired the design of JAK2/HDAC dual inhibitor **20a**.

This research emphasizes the therapeutic promise of JAK2/HDAC dual inhibitors in the treatment of AML and IFIs, offering an effective approach for multitargeted drug discovery. Building on the prior work of C. Sheng and his team, in multitarget drug design, a series of new JAK2‐HADC inhibitors were developed based on the synergistic interaction between the two targets (Meyer and Levine 2014). The pharmacophore of JAK2 inhibitors typically comprises a hinge‐region binder (usually an aminopyrimidine), two hydrophobic moieties (phenyl groups), and a solvent‐exposed component that functions as a template for the construction of dual inhibitors. The aminopyrimidine of the JAK2 inhibitor CYT‐387 (Figure 20) has been established as the determinant of JAK2 inhibitory activity; thus, this motif was preserved in the design of the dual inhibitors. HDAC inhibitors typically comprise a hydrophobic cap group, a linker (alkyl, alkenyl, or aryl), and a zinc‐binding group (ZBG), such as hydroxamic acid or benzamide (Gryder et al. 2012). Considering the cap group's flexibility and its potential for substitution with alternative moieties to produce HDAC inhibitors (Figure 20), the initial series of dual inhibitors were conceived by integrating the core group of CYT‐387 with the ZBG motif (hydroxamic acid or benzamide) via diverse linkers. Furthermore, based on the observation that *N*‐phenylmethanesulfonamide derivatives of compound CYT‐387 demonstrated comparable JAK2 inhibition, the subsequent series of JAK2‐HDAC inhibitors were formulated by substituting *N*‐cyanomethylbenzamide for *N*‐phenylmethanesulfonamide.

Molecular docking studies were conducted to examine the binding modes of compounds **20a**, **20h**, and **24** with JAK2 and HDAC1. Compound **20a** interacts with HDAC1 (PDB ID: 4BKX) (Millard et al. 2013) primarily via the linker and the zinc‐binding group (ZBG). The hydroxamic acid of **20a** coordinated with the catalytic Zn^2+^ of HDAC1, establishing two hydrogen bonds with Gly146 and His178, respectively. The hydrophobic cinnamamide moiety established π − π stacking interactions with His178, Phe150, and Phe205. Furthermore, the N‐phenylmethanesulfonamide moiety established three supplementary hydrogen connections with Glu146, Lys143, and Ser148.

With JAK2 (PDB ID: 4AQC) (Dugan et al. 2012), the aminopyrimidine framework of compound **20a** established hydrogen bonds with the backbone of Leu932 at the hinge region. The *N*‐phenyl‐methanesulfonamide moiety established an extra hydrogen bond with Asp994. Furthermore, the linker and ZBG protruded from the binding site and established hydrogen connections with Asp939 and Arg980, respectively.

#### 2.4.2 BRD4 + HDAC Inhibitors

Bromodomain extra‐terminal domain (BET) is a subfamily of BRDs that regulate gene transcription by binding to acetylated chromatin (Filippakopoulos and Knapp 2014). The BET family proteins BRD2, BRD3, BRD4, and BRDT each have two bromodomains (BD1 and BD2) (Filippakopoulos and Knapp 2014).

Due to their importance in *C. albicans* proliferation, survival, stress response, and virulence, BET proteins were also prospective antifungal targets (Mietton et al. 2017).

**FIGURE 21:**
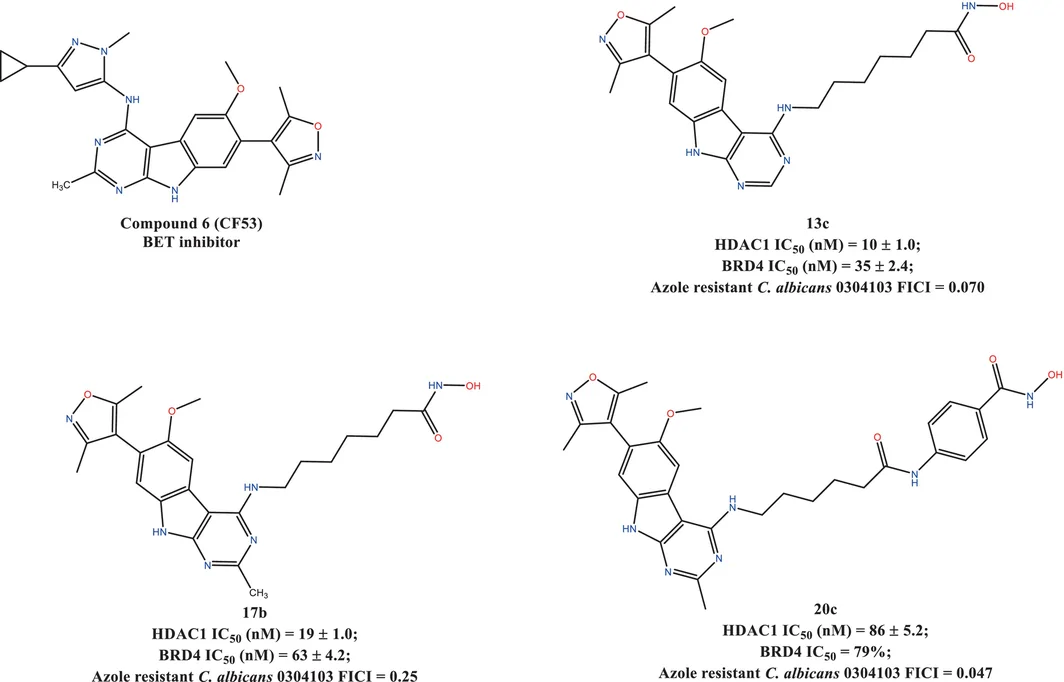
Dual inhibitor of BRD4 and HDAC.

Multiple chemotypes of pan‐BET inhibitors have been recognized, including azepines, dimethylisoxazoles, pyridones, triazolopyrazines, tetrahydroquinolines, 4‐acyl pyrroles, and 2‐thiazolidinones (Liu et al. 2017). The dimethylisoxazole scaffold demonstrated exceptional BET inhibitory efficacy and pharmacokinetic characteristics, serving as a promising foundation for the development of BET−HDAC dual inhibitors. Compound **6** was chosen as a template for dual inhibitor design due to its superior BRD4 inhibitory efficacy, clearly established structure–activity relationships (SARs), and binding manner (Zhao et al. 2018). The dimethylisoxazole moiety of compound **6** serves as a lysine acetylation (KAc) mimic and occupies the KAc binding pocket, indicating its essential involvement in BRD4 binding. Consequently, the dimethylisoxazole component was retained in the creation of dual inhibitors. The conventional pharmacophore model of most HDAC inhibitors comprises three components: a hydrophobic cap group that occupies the entrance of the binding pocket, a zinc‐binding group (ZBG) that chelates with the catalytic Zn^2+^ ion in the active site, and a hydrophobic linker that connects the two components. Considering the cap group's extensive adaptability to many structures, it is prudent to integrate the linker and hydroxamic acid group into the scaffold of BET inhibitor **6** (Figure 21). Furthermore, it has been shown that the incorporation of the methyl group into the tricyclic framework may enhance metabolic stability (Zhao et al. 2018). Consequently, the methyl‐substituted compounds were likewise synthesized. Ultimately, taking into account the diversity of the linker, compounds including alkyl‐aromatic linkers were also created.

Compounds **13c** and **17b** exhibited significant in vitro and in vivo anticancer and synergistic antifungal properties. In comparison to BET or HDAC inhibitors, the dual‐targeting inhibitors exhibited significant advantages, including enhanced efficacy against breast cancer and superior synergistic activity against *C. albicans* . This study enhanced the therapeutic potential of BET−HDAC dual inhibitors. Compounds **13c** and **17b** markedly reduced the fungal kidney load in the resistant candidiasis model when administered in conjunction with FLC. Consequently, they represent attractive lead drugs for the combinatorial therapy of breast cancer and concomitant candidiasis. It is noteworthy that other documented BET−HDAC dual inhibitors may have analogous actions. Additional pharmacological assessments, structural refinements, and mechanism investigations are under underway (Huang et al. 2023).

**FIGURE 22:**
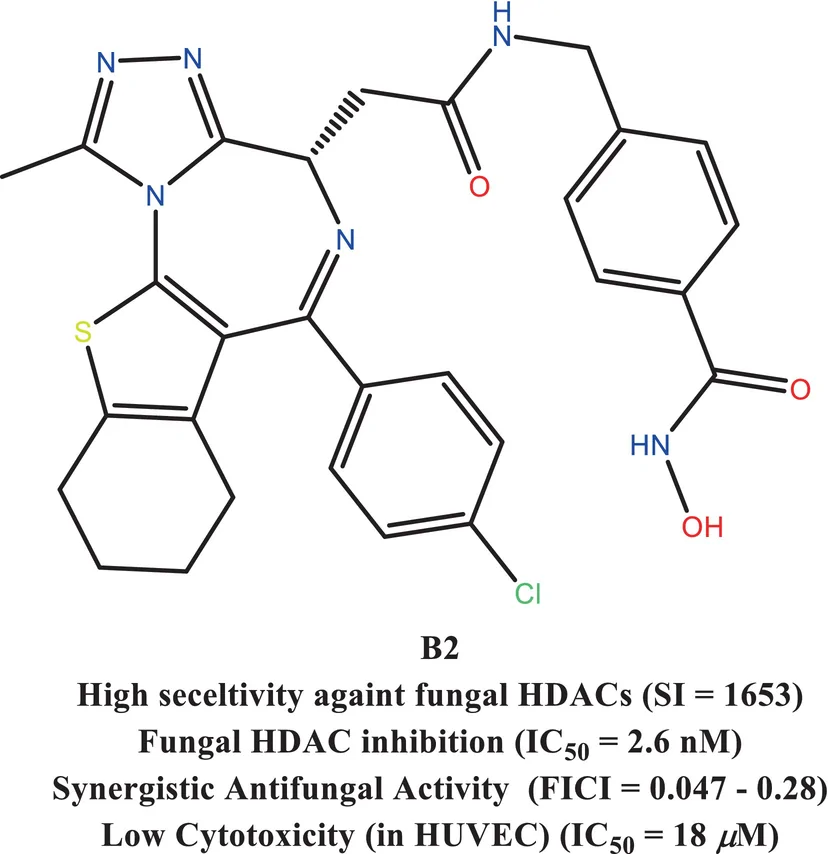
Compound that exhibited significant inhibitory action against HDAC1 and BRD4.

Another study involved the design and evaluation of a variety of novel BRD4 − HDAC inhibitors. The pharmacological assessments revealed that the target compounds did not demonstrate efficacy as anticancer agents. Conversely, several demonstrated significant selectivity towards fungal HDACs. Compound **B2** (Figure 22) exhibited significant inhibitory action against HDAC1 and BRD4. Compound **B2** exhibited remarkable selectivity against whole fungal cell HDACs, achieving a selectivity index of 1653, and demonstrated a potent synergistic impact with FLC against resistant *C. albicans* . The mechanistic studies indicated that the reversal of *C. albicans* resistance resulted from decreased expression of the azole target gene ERG11, efflux pump genes (CDR1, CDR2, and MDR1), and extracellular matrix‐related genes (GCA1 and ADH5) due to the drug combination. The downregulation effects can enhance the sensitivity of azoles to CYP51, augment the intracellular accumulation of pharmaceuticals, and diminish the barrier to drug entrance into cells. Furthermore, the medication combination obstructed morphological alterations and hindered fungal biofilm development, hence diminishing fungal pathogenicity. Significantly, compound **B2** enhanced the efficacy of FLC and substantially diminished the renal *C. albicans* load in a murine candidiasis model. Selective fungal HDAC inhibitors indicate a promising approach for addressing resistant invasive fungal infections. Ongoing efforts include more structural optimization, selectivity studies of fungal HDAC subtypes, and investigations into mechanisms (Li, Huang, et al. 2023).

### 2.5 Multifunctional, Miscellaneous Antifungal Agents

It is well known that the rise of aggressive, resistant, and swiftly changing fungal diseases presents a substantial risk to human health, agriculture, and the environment and intervening in cellular processes with conventional small molecules may be efficacious but necessitates prolonged development periods and is susceptible to antibiotic resistance. This study utilizes CRISPR‐Cas systems as antifungals to address the existing constraints in antibiotic creation and treatment, leveraging their adaptability, specificity, and efficiency in target design. The traditional design of CRISPR‐Cas antimicrobials, which relies on the creation of DNA double‐strand breaks (DSBs), may be less successful in fungi due to their resilient eukaryotic DNA repair mechanisms. Trinh and the team presented a novel design strategy for developing more powerful CRISPR‐Cas antifungals by simultaneously targeting essential genes and DNA repair genes, therefore incapacitating the fungi's capacity to repair double‐strand breaks in important genes. Through the assessment of this strategy on the model organism *Saccharomyces cerevisiae* , they established that the cotargeting of essential and defense genes is more efficacious than targeting either category of gene in isolation. The highest‐performing CRISPR‐Cas antifungals exhibited efficacy comparable to the antibiotic Geneticin (Mendoza et al. 2023).

An examination of gene cotargeting interactions demonstrated that the cotargeting of critical genes with RAD52, which is implicated in homologous recombination (HR), constituted the most synergistic combination. The rapid growth kinetics of *S. cerevisiae* elicited resistance to CRISPR‐Cas antifungals, predominantly resulting in genetic changes inside defense genes and guide RNA sequences.

The molecular structures Zhou, Lin, Lv and their colleagues aimed to create were chemically developed considering the following factors: The naphthalimido moiety, characterized by a polycyclic aromatic skeleton with two amido groups, diverges from traditional antifungal structures. This distinctive naphthalimido fragment serves as an antifungal scaffold with multitargeting capabilities, engaging biological targets through noncovalent interactions, thereby addressing the escalating issue of global drug resistance (Gong, Baathulaa, et al. 2016; Gong, Addla, et al. 2016).

The two carbonyl groups in the naphthalimido backbone can establish noncovalent interactions with biological targets, which are advantageous for increasing affinity towards these targets and modulating the physicochemical features of the engineered molecules.

A hydroxyethyl moiety is acknowledged as a valuable component in antimicrobial drugs, such as fluconazole, where it can engage in supramolecular interactions with biological target molecules through hydrogen bonding, thereby enhancing water solubility and bioavailability (Sun et al. 2022). Consequently, a hydroxyethyl moiety was incorporated into the target molecules. To examine the necessity of integrating a hydroxyethyl group into our target molecules, the hydroxyethyl fragment was substituted with a comparable hydrophilic amino group, capable of forming hydrogen bonds, along with many hydrophobic fragments such as methyl and ethyl groups.

Nitrofuran compounds demonstrate significant antibacterial efficacy against several pathogenic species. Their antimicrobial mechanism is ascribed to the free radical intermediates generated by the reduction of the 5‐nitro group by nitroreductases, such as nitrofurazone sensitivity protein B or azoreductases, which can provoke the generation of substantial quantities of reactive oxygen species (ROS) through redox cycling, thereby inducing oxidative stress and metabolic dysfunction, ultimately leading to apoptosis.

Moreover, the production of electrophilic intermediates such as nitroso and hydroxylamine might potentially impede bacterial DNA synthesis (Zhou et al. 2012). The nitro group, possessing a considerable electron‐withdrawing capacity, positively influences the pharmacological activity of certain medicines, including nitroimidazole, fexinidazole, and ranitidine. Activation by nitro reduction can lead to the generation of reactive oxygen species (ROS), potentially disturbing the glutathione (GSH)/oxidized glutathione (GSSG) ratio, so impacting the normal functionality of many cellular enzymes (Krasavin et al. 2018). To evaluate the significance of the nitro group, it was substituted with other groups, including hydrophobic methyl and hydrophilic hydroxymethyl substituents.

A hydrazyl group is a kind of Schiff base including two nitrogen atoms, capable of readily forming hydrogen bonds with biological molecules. It is inherently found in numerous antibacterial agents, including ftivazide, ceredon, and nitrofurazone. Additionally, nitrogen atoms in the hydrazyl group are susceptible to creating ionic interactions with acidic amino acid residues in biological molecules (Zhao et al. 2023). The hydrazyl group (−CH=N−NH−) has two nitrogen atoms, with one nitrogen atom forming a σ bond with the other nitrogen atom. This σ single bond is rotatable and flexible, enabling the molecule to adapt its conformation suitably for binding. The hydrazyl proton can be extracted by a base, resulting in the formation of the corresponding anion, whereas both hydrazine and its anion can be oxidized to generate the respective hydrazyl free radicals. Furthermore, the hydrazyl group can enhance the overall stability of nitrofuran when the nitro group generates free radicals, which also facilitates the generation of free radicals by nitro groups (Liu, Xi, Wang, et al. 2022; Shakir et al. 2017; Zuma et al. 2019).

The alkenyl group can influence molecular flexibility and modify stereochemical characteristics without altering charge distribution, thereby impacting the associated properties of pharmaceuticals. It is frequently employed for the rational optimization of compounds in pharmaceutical design (Tok et al. 2021). Employing the vinylogy rule, an alkenyl moiety was interposed between hydrazone and nitrofuran to synthesize a nitrofuryl hydrazylnaphthalimidol (HN) featuring an elongated linker for the assessment of its activity.

Thienyl‐based tioconazole was previously utilized as an antifungal agent in clinical therapy. The nitrothienyl group possesses a structure and chemical characteristics akin to those of nitrofuran (Peng et al. 2013). Consequently, the viability of nitrothienyl and unsubstituted thienyl groups as substitutes for nitrofuran was examined.

A thiazolyl group, such as a thienyl isostere, demonstrates favorable medicinal potential and is commonly utilized in the development of innovative antifungal medicines. Certain thiazole‐derived antifungal agents, including abafungin, ravuconazole, and fosravuconazole, have been utilized in clinical settings (Wang, Zhang, et al. 2021; Wang, Ansari, and Zhou 2021; Chen et al. 2017).

This study compares the contributions of several linkers between naphthalimide and thiazolyl groups to their activity. The substituents on the thiazolyl ring were examined as well.

The pyrrolyl ring possesses a single hydrogen bond donor, enhancing its affinity for biological targets, and is thus extensively utilized in the creation of pharmaceutical compounds such as kainic acid and lincomycin (Narva et al. 2016).

A study led by Jing‐Song Lv, Jian‐Mei Lin, and Cheng‐He Zhou employed a pyrrolyl ring with various substituents, including isosteres, to substitute the furyl ring and examine their impact on activity.

The imidazolyl group is a significant functional moiety containing two nitrogen atoms, commonly found in various biomolecules such as histidine and histamine, as well as in antifungal agents. Its distinctive structure readily associates with diverse biological targets via noncovalent interactions, thereby surmounting drug resistance, augmenting bioavailability and biocompatibility, and amplifying efficacy (Li, Tan, et al. 2023; Dai et al. 2023; Li, Zhang, et al. 2022). Imidazoles, including clotrimazole, miconazole, and ketoconazole, are early antifungal agents that remain extensively utilized in clinical settings. The limited antifungal spectrum of imidazole medicines underscores the imperative to create novel broad‐spectrum antifungal medications. The impact of the imidazolyl ring with diverse substituents as isosteres of the pyrrolyl group on antifungal activity was examined.

A phenyl ring is frequently employed as an isostere of the thienyl group in pharmaceutical development; numerous successful instances have been documented, such as the clinical antifungal tioconazole, where its thienyl moiety is substituted with a phenyl group to yield the antifungals econazole and sulconazole. Furthermore, phenols can denature microbial proteins and disrupt the plasma membrane, demonstrating an antibacterial action (Li, Zhao, et al. 2022).

**FIGURE 23:**
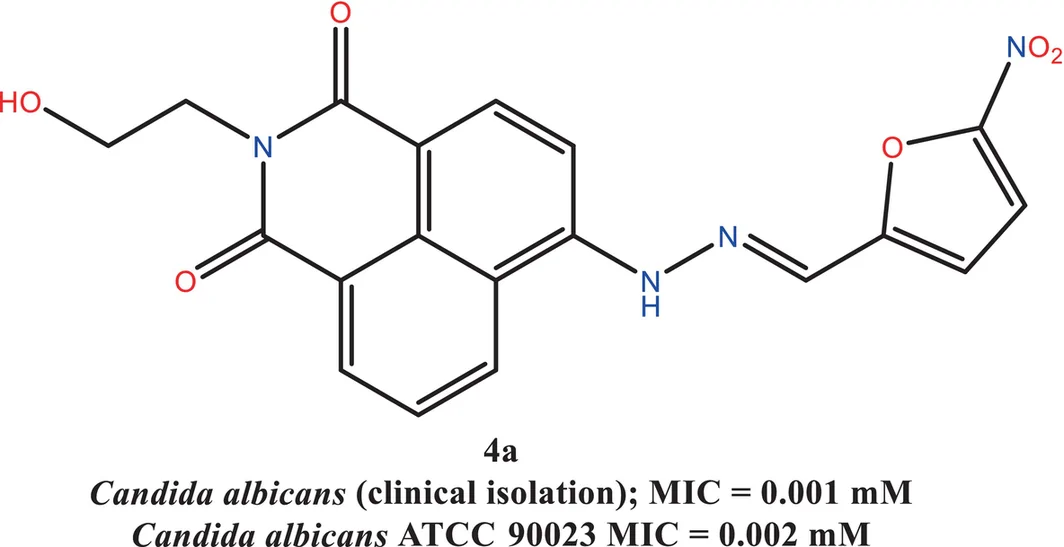
Nitrofuryl multitargeting anti‐fungal agent.

Consequently, several hydroxyphenyl compounds and their equivalents were introduced to assess their role in antifungal activity. Pyridiniums containing positive ions are frequently identified in many natural products and antibacterial treatments, including cetylpyridinium chloride, utilized in formulations for managing supragingival plaque and gingivitis (Sowmiah et al. 2018). These lipophilic cations can modify the membrane potential of the cell membrane, impairing their function and exhibiting antifungal activity. Consequently, various substituted pyridiniums were introduced to examine their impact on activity. A distinctive category of HNs was produced in high quantities as potential antifungal drugs through efficient synthetic pathways. All the novel compounds were studied using ^1^H NMR, ^13^C NMR, and HRMS spectroscopy. The X‐ray single‐crystal diffraction investigation verified that the geometry of 4‐chlorobenzyl imidazolyl HN is in a trans‐configuration. Certain target compounds demonstrated significant antifungal efficacy. Nitrofuryl HN **4a** (Figure 23) exhibited superior activity with a MIC value of 0.001 mM compared to the therapeutic fluconazole (MIC = 0.013 mM) against *C. albicans* . Moreover, nitrofuryl HN **4a** exhibited a swift fungicidal efficacy, minimal resistance propensity, low cytotoxicity, a safe hemolysis threshold, advantageous drug‐likeness, and the capacity for transport by HSA. The biofunctional investigations indicated that nitrofuryl HN **4a** could induce shrinking of the *C. albicans* surface, although it does not directly cause membrane damage. Moreover, it may decrease LDH activity, elevate ROS levels, and intercalate into DNA, resulting in metabolic inactivation, oxidative stress, GSH depletion, and obstruction of DNA replication.

Furthermore, nitrofuryl HN **4a** exhibits a low energy gap value and a strong molecular electrostatic potential, which may facilitate bond formation with the targets. The results indicated that nitrofuryl HN **4a**, as a new multitarget antifungal drug, possesses considerable advantages in addressing fungal infections and shows tremendous promise for further research (Wang et al. 2024).

## 3 Summary & Conclusion

For therapeutic use, novel antifungals that inhibit drug‐resistant microorganisms are the need of the hour. The pharmaceutical industry followed the “one molecule, one target, one disease” philosophy throughout the 20th century. This idea has produced several effective selective medications that will dominate disease treatment in the future. In complex *Candida* infection, manipulating a single target may not always work, even with the best research. Thus, medicines that activate several targets to improve efficacy and safety are attracting attention.

It is considered to be a smarter approach to employ MTDLs that target pathogenesis at multiple sites (biomolecules like proteins/nucleic acids or cell membrane etc.) to treat multifactorial illnesses. Apart from the well‐known advantages, the notable disadvantages of MTDLs are as follows: (1) the difficulty of simultaneously optimizing two pharmacophores, (2) the effect of increased molecular weight on drug‐likeness and pharmacokinetics, (3) the difficulty of balancing potency across targets, and (4) off‐target toxicity arising from promiscuity etc.

We believe that multitarget drug design will take a more systematic and thorough approach to target combinations, ligand selection, and desired activity equilibrium to optimize developability, effectiveness, and safety of antifungal agents in near future. This review discusses major difficulties, investigates antifungal multitarget drug design, and surveys new, promising MTDL references to provide a comprehensive, comparative compendium of rational MTDL design to help the budding researchers in this field of drug design.

## Author Contributions

**Iftakhar Hussain Ansari:** writing – review and editing. **Sarmistha Pal:** conceptualization, funding acquisition, writing – original draft, writing – review and editing, supervision, visualization. **Sriparna Pusti:** writing – review and editing, visualization. **Hritabrata Bhattacharya:** writing – review and editing. **Shuvradip Das:** writing – review and editing, visualization. **Sanchita Pradhan:** writing – review and editing.

## Funding

The study is supported by the seed funding by Research Funding & Management Office (RFMO), JIS Group of Institutions (RFMO/SF/JISU/24‐25/002).

## Conflicts of Interest

The authors declare no conflicts of interest.

## Data Availability

Data sharing is not applicable to this article as no data sets were generated or analyzed during the current study.
